# Twelve Fused Models by Fusing Four Types of Transformer-Based Tuners on Three Base UNet Architectures for Carotid Wall Segmentation and Plaque Burden/Intima-Media Thickness Measurements in Ultrasound Scans: A Scientific Validation Study

**DOI:** 10.3390/diagnostics16162538

**Published:** 2026-08-12

**Authors:** Nikhil Singh, Rubeena Vohra, Arun K. Dubey, Laura E. Mantella, Amer M. Johri, Esma R. Isenovic, John Laird, Mustafa Al-Maini, Vijay Viswanathan, Mohamed Abbas, Gavino Faa, Janet N. A. Ajuluchukwu, Andrew Laine, Luca Saba, Jasjit S. Suri

**Affiliations:** 1 Department of Electronics and Communication Engineering, Bharati Vidyapeeth’s College of Engineering, New Delhi 110063, India; aixnick@icloud.com (N.S.);; 2 Stroke Monitoring and Diagnostic Division, AtheroPoint™, Roseville, CA 95661, USA; 3 Department of Computer Science Engineering, Samrat Ashok Rajkiya Engineering College (SAREC), New Delhi 23100, India; 4 Division of Cardiology, Department of Medicine, Queen’s University, Kingston, ON K7L 3N6, Canada; 5 Department of Biomedical and Molecular Sciences, Queen’s University, Kingston, ON K7L 3N6, Canada; 6 Department of Radiobiology and Molecular Genetics, “VINČA” Institute of Nuclear Sciences—National Institute of the Republic of Serbia, University of Belgrade, 11001 Belgrade, Serbia; 7 Department of Cardiology, St. Helena Hospital, St. Helena, CA 94574, USA; 8 Allergy, Clinical Immunology and Rheumatology Institute, Toronto, ON M5G 1N8, Canada; 9 MV Diabetes Center, Chennai 600013, India; 10 Electrical Engineering Department, College of Engineering, King Khalid University, Abha 61421, Saudi Arabia; 11 Department of Pathology, University of Cagliari, 09124 Cagliari, Italy; 12 Department of Biomedical and Radiology, Columbia University, New York, NY 10027, USA; 13 Department of Radiology, Azienda Ospedaliero-Universitaria (A.O.U.) di Cagliari, 09124 Cagliari, Italy; 14 Department of Electrical and Computer Engineering, Idaho State University, Pocatello, ID 83209, USA; 15 Symbiosis Institute of Technology, Symbiosis International (Deemed University), Nagpur Campus, Pune 440008, India

**Keywords:** carotid ultrasound, wall segmentation, UNet, transformer-based tuners, scientific validation, variability analysis

## Abstract

**Background/Objectives:** UNet-based models dominate medical image segmentation. Transformers have been added as an internal variant to these UNet-based architectures to improve feature learning. However, they have limitations in generalization and computational efficiency. Motivated by this idea, we have designed a two-stage novel hybrid segmentation framework, where tuners are added in cascade to the base architectures. **Methods:** Three sets of base UNets were designed, namely: B1: UNet1p, B2: UNet2p, and B3:UNet3p, and four sets of transformer-based tuners were designed, namely: T1:Transformer-augmented UNet, T2: Attention-guided UNet, T3: Swin Transformer-based UNet, and T4: Pyramid-based network, leading to 12 fused systems that combine three base UNets and four Tuners, namely: F1: B1 + T1, F2: B1 + T2, F3: B1 + T3, F4: B1 + T4; F5: B2 + T1, F6: B2 + T2, F7: B2 + T3, F8: B2 + T4, F9: B3 + T1, F10: B3 + T2, F11: B3 + T3, F12: B3 + T4. **Results:** The two-hybrid segmentation models are more effective and reliable than the single-stage UNet architecture. B3 + T4 achieved a Dice of 94.14% and Jaccard of 88.7%, surpassing prior baselines by 4.2% and 6.8%. It reduced cIMTE to 0.014 mm, a 36% improvement and the lowest reported to date, with cLIE and cMAE errors lowered by 40%. **Conclusions:** All 12 hybrid automated transformer-based models are highly accurate and reliable for wall segmentation in carotid ultrasound; they are a powerful paradigm for cardiovascular risk.

## 1. Introduction

Atherosclerotic cardiovascular disease (CVD) represents a leading contributor to the global burden of disease, accounting for approximately five million stroke-related deaths annually and contributing substantially to worldwide morbidity and mortality. Its clinical impact is further amplified in patients with coexisting conditions, including renal disease [1,2], rheumatoid arthritis [3,4,5], prostate cancer, diabetic retinopathy [6], neurological diseases [7,8], peripheral artery diseases [9], and immunological disorders. Although imaging modalities like magnetic resonance imaging (MRI), computer tomography (CT), and intravascular ultrasound (IVUS) [10] are used to diagnose coronary artery diseases (CAD), they are either expensive or invasive. Further, they involve radiation exposure [11], minimally invasive [12] and not cost-effective [13] approaches. The carotid artery is genetically similar to the coronary artery and can be utilized to assess CVD risk [14,15,16]. Carotid Intima-Media Thickness (cIMT) is a widely accepted marker of CVD risk [17]. Previous studies have associated cIMT values above 1 mm with increased stroke risk and coronary atherosclerosis [18,19,20,21]. However, it is difficult to obtain consistent cIMT measurements because of anatomical variability and manual handling of ultrasound probes [22,23]. To address these challenges, we investigated four novel transformer-tuner architecture cascaded to three kinds of base UNet architectures: B1: UNet1p, B2: UNet2p, and B3: UNet3p, aimed at enhancing segmentation performance.

*Contributions:* This work’s contributions are: (1) a decoupled two-stage cascaded architecture in which coarse boundary localization (Stage I) and global contextual refinement (Stage II) are optimized as separable, interchangeable modules rather than fused in a single jointly trained network; (2) a systematic factorial evaluation of the full three-base × four-tuner space, disentangling base and tuner contributions rather than confounding them in a fixed hybrid; and (3) measurement-level and cross-domain validation, assessing models by clinical error in millimetres (cIMT, LI, MA, plaque area) under a cross-institutional, cross-ethnic unseen-domain protocol, not by Dice/Jaccard alone.

The paper is organized as follows: Section 2 reviews the related work. Section 3 presents the dataset and methodology. Section 4 reports the experimental results. Section 5 evaluates performance. Section 6 covers scientific validation and software verification. Section 7 presents the comparative analysis; Section 8 conducts the variability analysis. Section 9 reports the statistical tests. Section 10 addresses explainability and Section 11 provides the critical discussion, followed by the conclusion in Section 12. A complete list of abbreviations is provided in Appendix A.

## 2. Background Literature

Carotid Intima-Media Thickness (cIMT) is an established, non-invasive marker of subclinical atherosclerosis and a predictor of future cardiovascular and cerebrovascular events [15]. Because cIMT is defined by the distance between the lumen–intima (LI) and media–adventitia (MA) interfaces, its automated quantification is reduced to a boundary-delineation problem on B-mode ultrasound, where accuracy is limited by speckle noise, low LI/MA contrast, jugular-vein interference, and operator-dependent probe positioning. A large body of methodological work has therefore targeted reproducible LI/MA segmentation, and several comprehensive reviews have catalogued the evolution of these techniques [24].

Early automated pipelines relied on classical image-processing operators: intensity thresholding and morphological post-processing, edge-based operators, multiscale dynamic programming, and Hough-transform initialization to localize the arterial walls [25]. Deformable models became the dominant paradigm of this era, with active-contour (“snake”) formulations evolving LI and MA contours toward the interfaces under image-derived energies [26]. To prevent the two contours from collapsing or diverging, dual-snake and constrained-snake systems were introduced, coupling the LI and MA evolutions within a unified energy functional and, in some cases, first-order absolute-moment external energies for stable deformation. Although these methods established strong clinical baselines, they typically required careful parameter tuning, hand-crafted initialization, and manual quality control, and they degraded on low-contrast or plaque-laden scans [25].

The introduction of convolutional neural networks (CNNs) shifted the field toward learned, end-to-end segmentation. The encoder–decoder U-Net, with its symmetric skip connections that re-inject high-resolution spatial detail, became the reference architecture for carotid wall and plaque segmentation and was rapidly adapted through encoder–decoder variants for IMC delineation and cIMT measurement [17]. Subsequent redesigns of the skip pathway nested, densely connected skips in UNet++ [27] and full-scale skip connections in UNet 3+ [28] improved multi-scale feature fusion, while attention-gated U-Nets suppressed irrelevant activations to sharpen boundary localization and plaque segmentation for stroke-risk stratification. Multi-stage- and nested attention-guided designs, such as the NAG-Net cascade, further refined the LI/MA interfaces by decoupling region localization from boundary extraction [29]. Despite these gains, purely convolutional receptive fields remain fundamentally local, limiting the modelling of long-range anatomical context along the vessel wall in noisy ultrasound [30].

Transformers address this limitation through self-attention, which captures global dependencies directly. Following the Vision Transformer [30], hybrid CNN–transformer architectures were introduced to combine convolutional locality with transformer context, and recent surveys document their rapid adoption across medical image segmentation [31]. Representative single-stage designs include TransUNet, which embeds transformer blocks in the U-Net bottleneck [32]; Swin-UNet, which replaces convolutions with windowed shifted-window self-attention throughout the encoder–decoder [33]; and pyramid-vision-transformer backbones that reintroduce multi-scale hierarchy into the attention mechanism [34]. In the carotid domain specifically, transformer-based and attention-fusion models have recently been applied to joint intima-media and lumen segmentation and to plaque quantification, reporting competitive overlap and clinical-error metrics [35,36,37]. Our own prior framework, SSwAttSkip-XmerBot-UNet, integrated transformer blocks in the bottleneck with attention-guided skip connections for Japanese cohort carotid segmentation [38].

However, these state-of-the-art hybrids share two limitations that motivate the present work. First, by embedding transformer modules *inside* a single jointly trained encoder–decoder, they entangle boundary-level feature extraction with global contextual refinement, so the two functions cannot be optimized, swapped, or ablated independently [37,39,40]. Second, most are validated on a single cohort using overlap metrics (Dice, Jaccard) and are rarely tested for cross-institutional or cross-ethnic generalization, despite well-documented domain shift across scanners and populations [41]. Robust, clinically deployable cIMT estimation requires both architectural decoupling and measurement-level, cross-domain validation of the gap this study targets through a two-stage cascaded base UNet + transformer-tuner framework evaluated by millimetric clinical error on unseen cohorts [42].

## 3. Methodology

Transformer architectures model long-range contextual dependencies effectively, whereas UNet architectures preferentially encode low-level spatial and localisation cues that are essential for precise boundary delineation. Exploiting this complementarity, three baseline segmentation architectures (Base 1, Base 2, Base 3) was cascaded with four transformer-tuner modules (Tuner 1 to Tuner 4) to strengthen contextual feature learning while preserving spatial fidelity. Each tuner was paired with each baseline, yielding twelve fused configurations, which permitted a systematic factorial assessment of vessel-wall segmentation performance in carotid ultrasound.

### 3.1. Data Acquisition and Ethics Approval

#### 3.1.1. Database 1: Japanese Diabetic Common Carotid Artery Database

The DB1 dataset consisted of 379 CCA images from 147 male and 43 female patients (mean age: 68.78 ± 10.88 years), obtained at Toho University, Japan. Clinical markers included HbA1c 6.22 ± 1.04 mg/dL, LDL 101.59 ± 31.03 mg/dL, HDL 51.05 ± 14.56 mg/dL, and total cholesterol 175.39 ± 36.03 mg/dL. Of 190 patients, 73 were smokers, and 116 had hypertension, with a mean systolic/diastolic BP of 134.06 ± 9.14/87.03 ± 4.57 mm Hg.

##### Ethics Approval for the Japanese Database

This research was carried out in compliance with the Declaration of Helsinki (as revised in 2013). For the Japanese database, the Institutional Review Board of Toho University, Japan (Ohashi Ethics Committee, Authorization No. 13–56) approved the study, and written informed consent was obtained from all participants.

#### 3.1.2. Database 2: Hong Kong Post-Menopausal Women Common Carotid Artery Database

The DB2 consisted of 300 CCA images acquired from 50 Chinese post-menopausal women, aged 54–67 years (6 images/patient). A total of 28 patients (56%) had comorbidities: 1 diabetic, 3 hypertensive, 7 hypercholesterolemic, 15 with both hypertension and hypercholesterolemia, and 2 with all three abnormalities. Normal BP, cholesterol and fasting glucose levels were seen in the remaining 22.

##### Ethics Approval for the Hong Kong Database

The Hong Kong cohort was obtained from the dataset reported by Ruby et al. (2009) [43], where the study had already received ethical approval. The following statement was used as stated in [43]: “Eligible subjects were invited for clinical assessment and carotid ultrasound measurements. All participants gave written informed consent, and the study was approved by the Ethics Committee of the Chinese University of Hong Kong”.

### 3.2. Architecture

**Figure 1:**
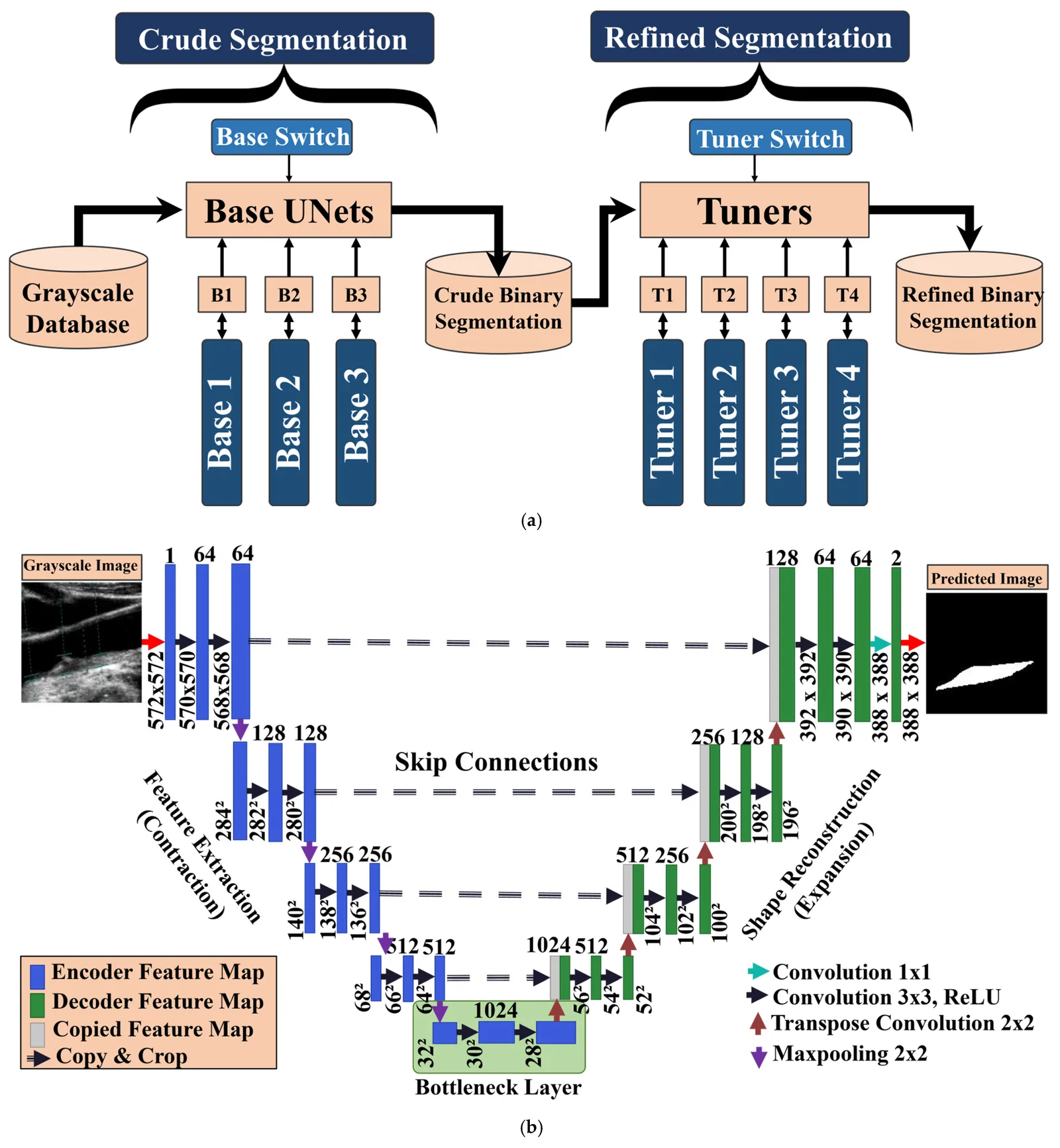

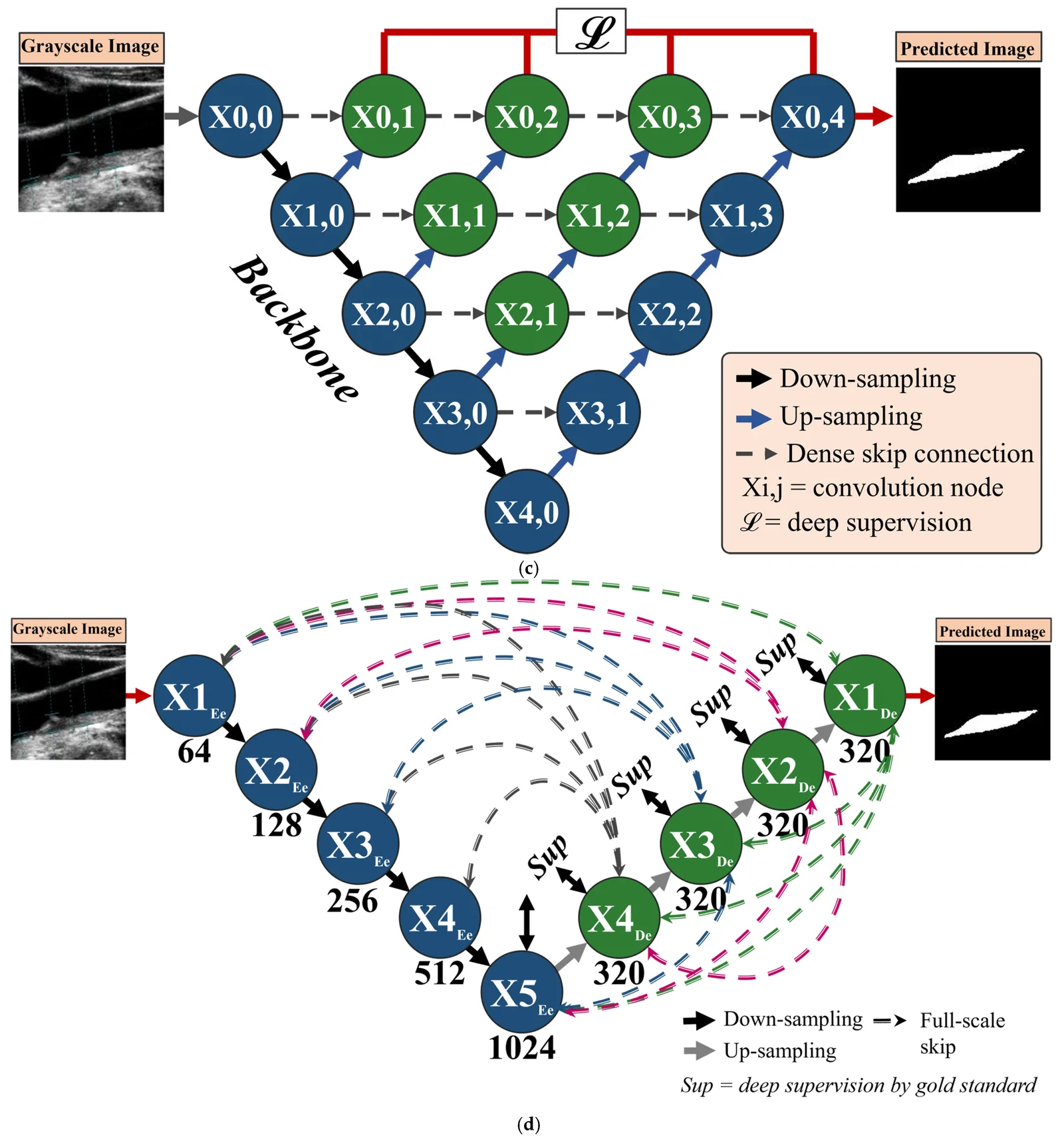
(**a**) Global overview of the hybrid segmentation approach. (**b**) Architectures of Base 1. (**c**) Architectures of Base 2. (**d**) Architectures of Base 3.

The proposed two-stage framework is shown in Figure 1a: a grayscale carotid scan first passes through a Stage I base UNet to produce a coarse vessel-wall mask, which together with the original image is refined by a Stage II transformer tuner to yield the final lumen–intima (LI) and media–adventitia (MA) boundaries used for cIMT and plaque-burden estimation.

*Stage I: Base networks:* (Figure 1b–d). B1 (UNet1p) is a conventional encoder–decoder UNet for low-level spatial feature extraction [44] (Figure 1b).

B2 (UNet2p) adds dense-nested skip connections for multi-scale aggregation [27] (Figure 1c).

B3 (UNet3p) uses full-scale skip connections to fuse features across all resolutions [28] (Figure 1d).

**Figure 2:**
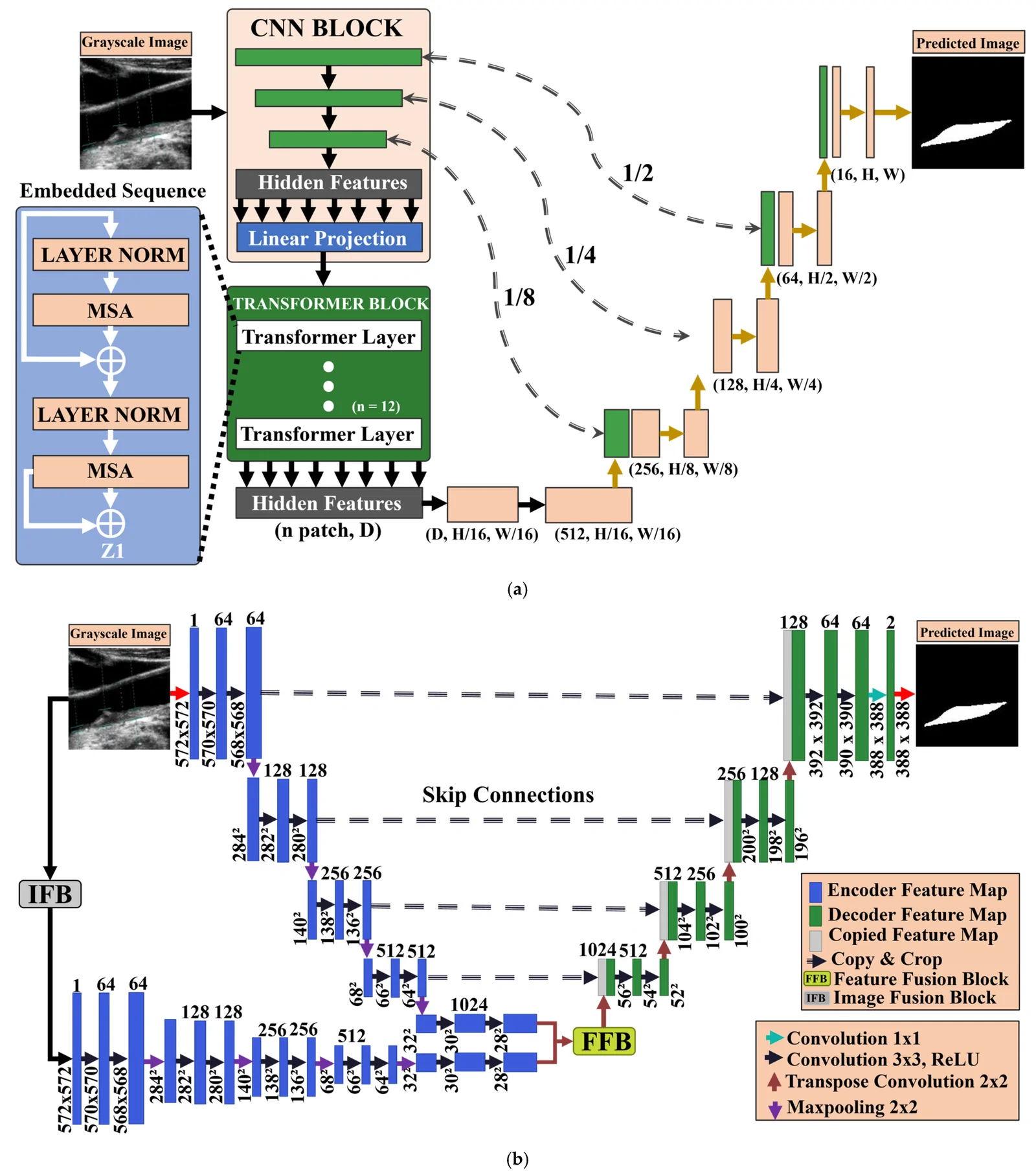

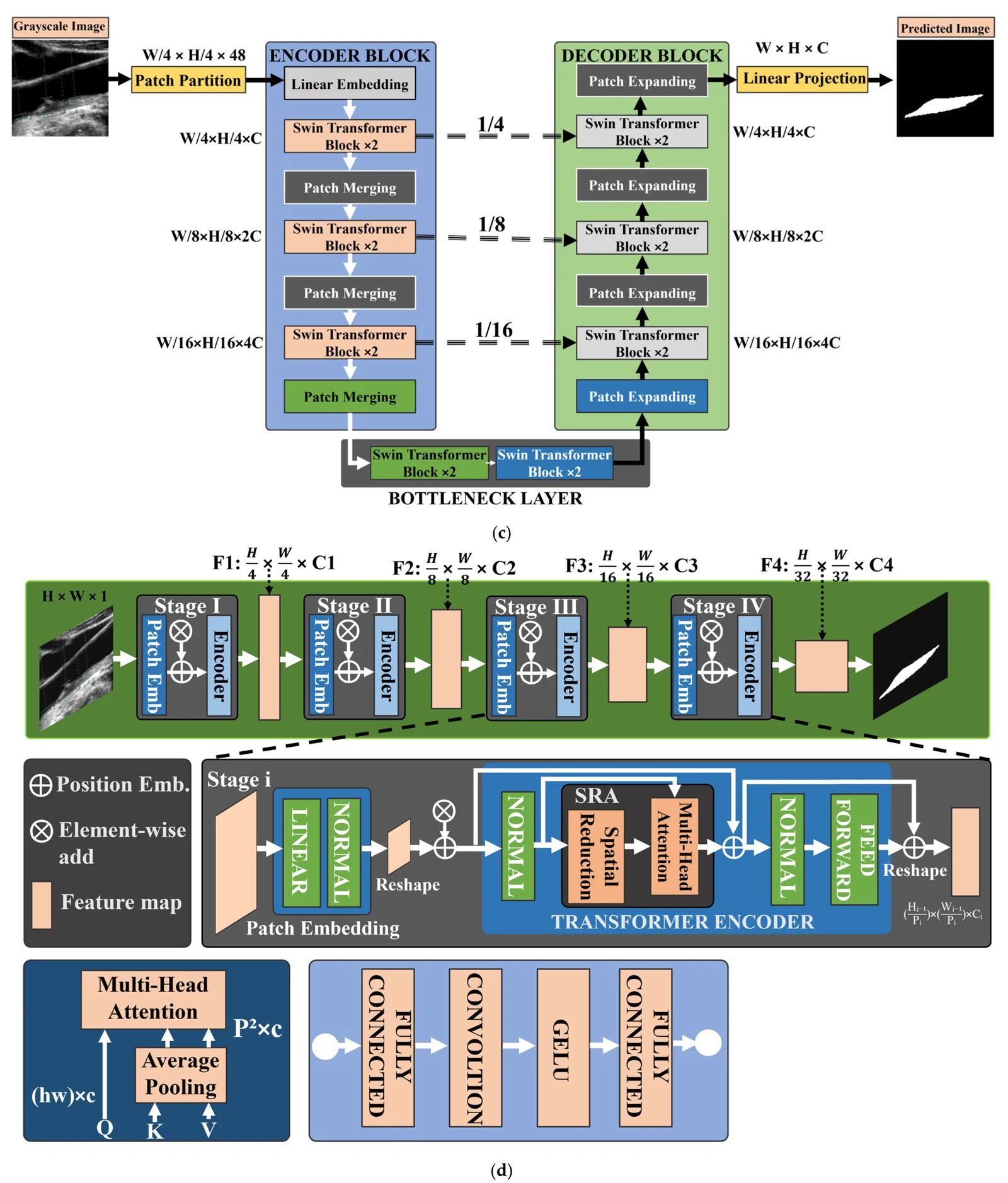
(**a**) Architecture of Tuner 1. (**b**) Architecture of Tuner 2. (**c**) Architecture of Tuner 3. (**d**) Architecture of Tuner 4.

Each base is cascaded with each tuner, yielding twelve fused models F1–F12 (F1: B1 + T1 … F12: B3 + T4). Full layer-level configurations of all base and tuner modules are provided in the Appendix B. *Stage II Transformer tuners:* (Figure 2a–d). T1 couples UNet features with transformer encoders for joint local–global modelling (Figure 2a).

T2 uses dual encoder pathways to align the original image with the Stage I mask [29] (Figure 2b). T3 applies Swin-windowed self-attention for long-range modelling [33] (Figure 2c). T4 integrates pyramid pooling within a transformer backbone for multi-scale boundary refinement [34] (Figure 2d). Unlike single-stage hybrids such as TransUNet [32] and Swin-UNet, which embed transformer blocks inside the encoder–decoder, the proposed design decouples boundary-level feature extraction (Stage I) from global contextual refinement (Stage II), allowing each stage to be optimized independently and any base–tuner pair to be interchanged without retraining the other. The complete cascaded base-tuner segmentation procedure, covering both Stage I and Stage II, is summarized Algorithm S1 (Supplementary Materials).

### 3.3. Experimental Protocol

*Experiment #1: Effect of Base Stage:* We first evaluated all base models independently under identical training conditions to establish performance baselines. This isolated the intrinsic segmentation capacity of each architecture and exposed its individual strengths and failure modes on carotid ultrasound. These baselines serve as the reference against which all subsequent augmentation, tuning, and fusion gains are measured.

*Experiment #2: Impact of Data Augmentation:* We applied geometric (rotations, flips, zooms) and photometric (intensity, brightness/contrast) transformations on the fly to simulate acquisition variability across operators and machines. Each model was trained with and without augmentation to isolate its marginal effect, with validation and test sets left untouched to avoid leakage. This quantifies how much augmentation alone narrows the cross-cohort domain gap.

*Experiment #3: Effect of Tuner Stage:* We introduced a tuning phase, performing systematic hyperparameter optimization (learning rate, weight decay, batch size, dropout) under an identical search budget for every model. Pre- and post-tuning metrics are reported to make the incremental gain explicit. This clarifies how much of each model’s ceiling is architectural versus an artefact of sub-optimal configuration.

*Experiment #4: K-Parameter Sensitivity:* We studied the effect of the parameter K by varying it across [K = 2, 3, 5, 10, TT] and recording the mean and standard deviation of performance for each setting. This confirms the reported gains are not tied to a particular data partition and identifies the K offering with the best accuracy–stability trade-off.

### 3.4. Loss Equation and Optimization

In this study, we adopted the binary cross-entropy (BCE) loss, which is well-suited to the binary (foreground–background) segmentation task of separating the target region from the surrounding tissue on a per-pixel basis. For each pixel, the loss penalizes the divergence between the predicted probability and the ground-truth label, and is expressed as:

(1) $$BCE\;Loss=\;-(\frac{1}{S})\times \sum \left(q_{i}\times \log\left({\hat{q}}_{i}\right)+\left(1-q_{i}\right)\times \log\left(1-{\hat{q}}_{i}\right)\right)$$

With $q_{i}$ being a true binary label (0 or 1), ${\hat{q}}_{i}$ represents the predicted probability obtained through a sigmoid activation, and S is the number of samples.

### 3.5. Implementation Details

All models were implemented in Python 3.14 with PyTorch 2.11.0 + cu128 and trained on an NVIDIA L40S GPU (NVIDIA Corporation, Santa Clara, CA, USA) via the Vast.ai cloud GPU platform (Vast.ai Inc., San Francisco, CA, USA). Networks were optimized with the Adam optimiser at an initial learning rate of 1 × 10^−4^, chosen for its adaptive per-parameter updates and stable convergence on medical-imaging tasks, a batch size of 16 and 200 epochs, with all images and masks resized to 256 × 256 pixels. Data augmentation was applied on-the-fly and comprised horizontal flipping, random rotation (±15°), slight tilting (±10°), brightness and contrast jittering (±10%), Gaussian noise injection, and elastic deformation constrained to preserve the anatomical plausibility of the LI and MA boundaries.

## 4. Results

### 4.1. Lumen–Intima Media–Adventitia Borders Visualization

**Figure 3:**
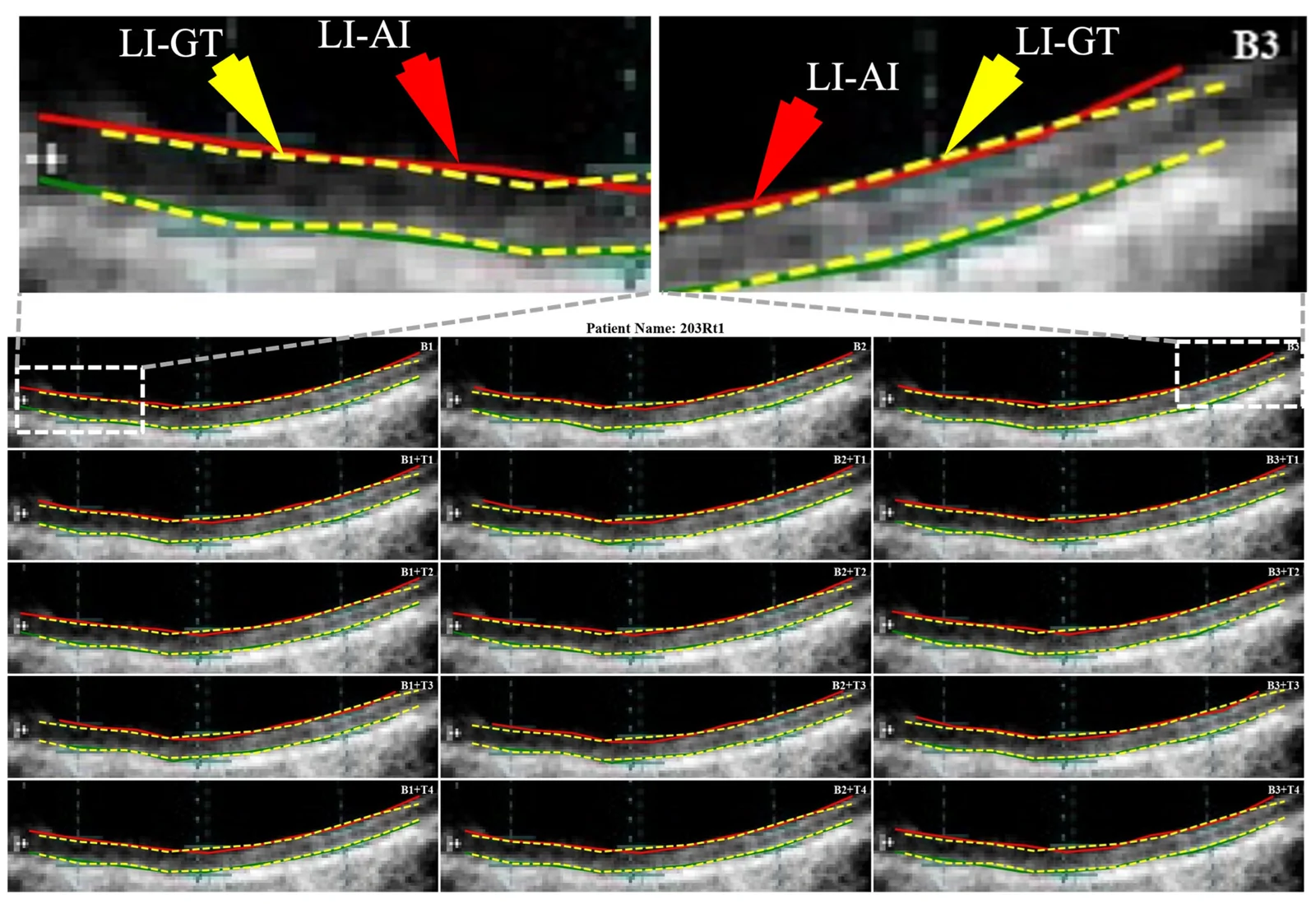
LIMA Border Visualization by three base and four tuner combination (data, Ethics approved, Courtesy of Toho University, Japan).

The Lumen–Intima Media–Adventitia borders (LIMAs) borders are depicted in Figure 3 below for each models, along with the segmentation accuracy for the LI and MA borders separately. In these images, the yellow line represents the ground truth, red markings indicate the LI border, and green markings indicate the MA borders. The region enclosed between these two borders represents the Intima-Media Thickness (IMT) area. The detailed computation formulas are provided in Appendix C.

### 4.2. Seen and Unseen Analysis Visualizations

**Figure 4:**
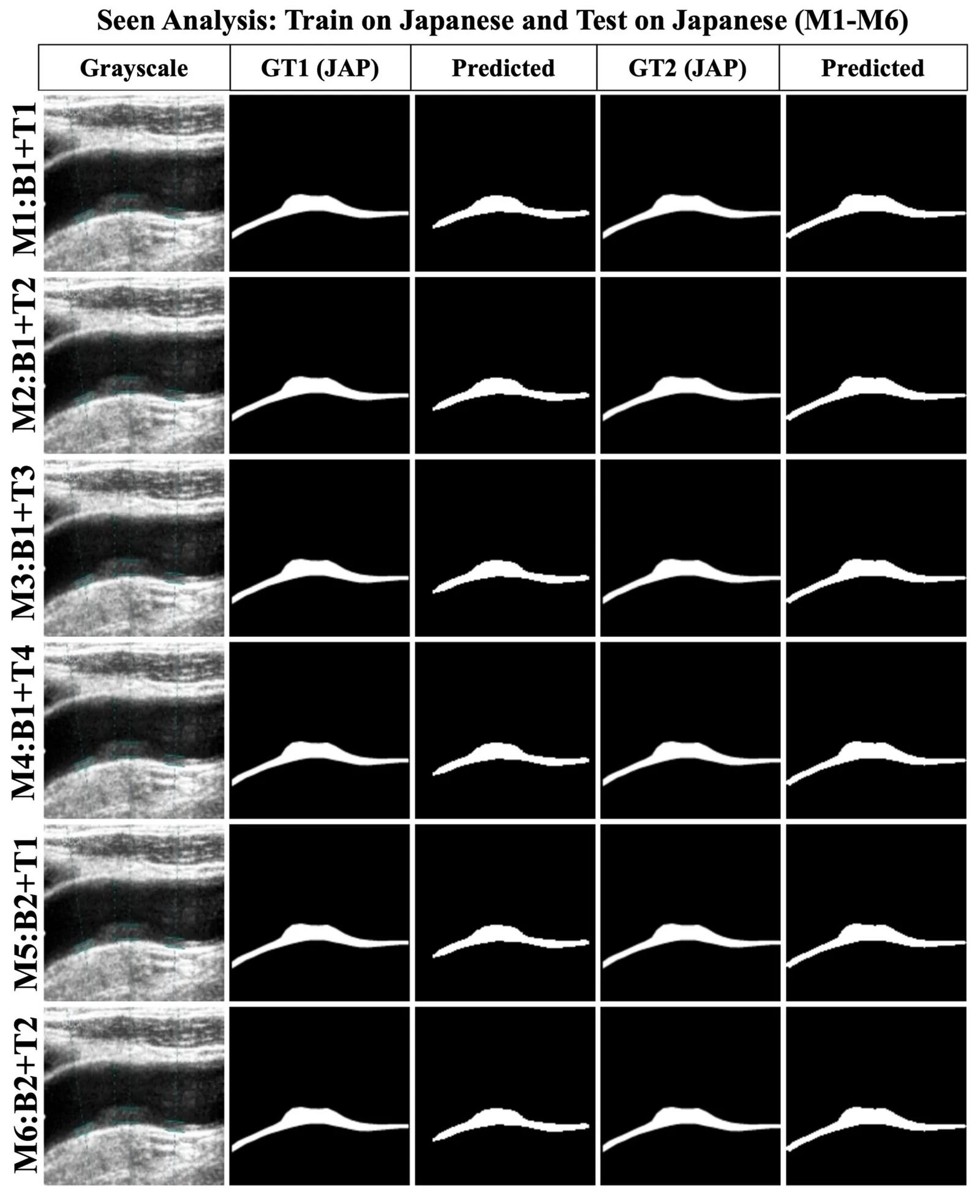
Seen-domain segmentation performance where the model is trained and evaluated on the Japanese dataset (M1–M6).

Figure 4 illustrates the qualitative results for the seen-domain analysis, where the model is trained and tested on the Japanese dataset. The figure shows grayscale inputs, corresponding ground-truth masks (GT1 and GT2), and predicted segmentation outputs for Models 1–6.

**Figure 5:**
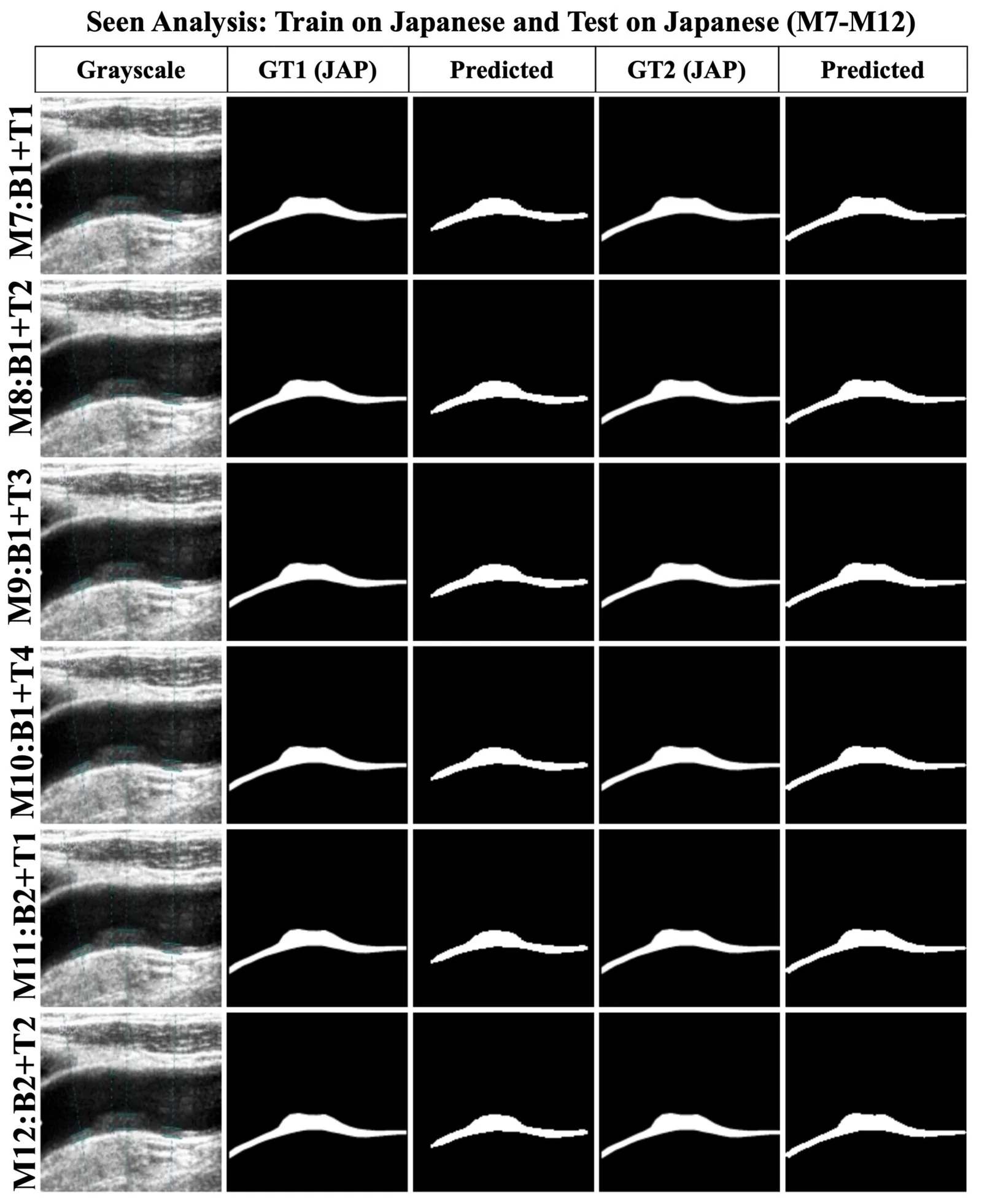
Seen-domain segmentation performance where the model is trained and evaluated on the Japanese dataset (M7–M12).

Figure 5 illustrates qualitative results for the seen-domain analysis, in which the model is trained and tested on the Japanese dataset. The figure shows grayscale inputs, corresponding ground-truth masks (GT1 and GT2), and predicted segmentation outputs for Models 7–12.

**Figure 6:**
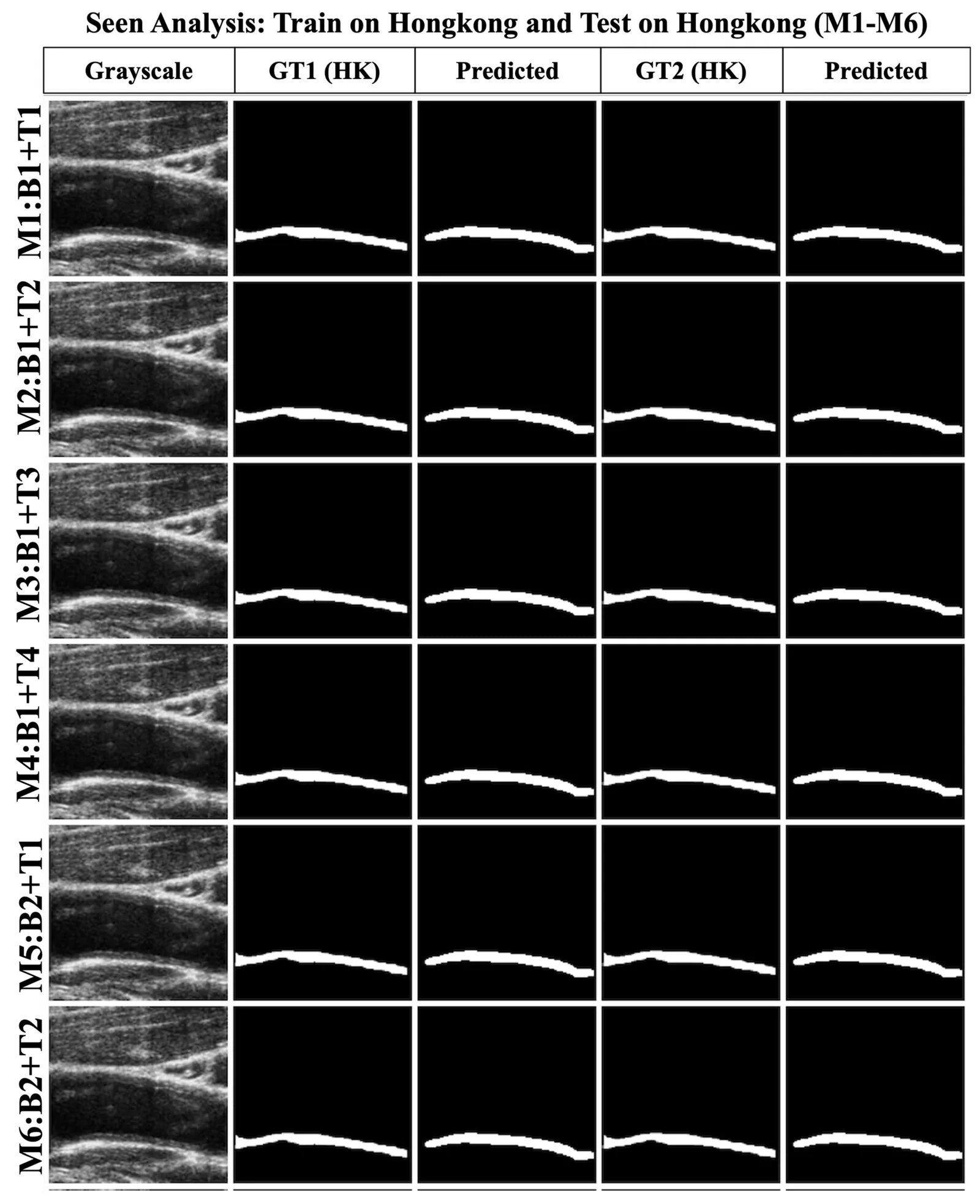
Seen-domain segmentation performance where the model is trained and evaluated on the Hong Kong dataset (M1–M6).

Figure 6 illustrates qualitative results for the seen-domain analysis, in which the model is trained and tested on the Hong Kong dataset. The figure shows grayscale inputs, corresponding ground-truth masks (GT1 and GT2), and predicted segmentation outputs for Models 1–6.

**Figure 7:**
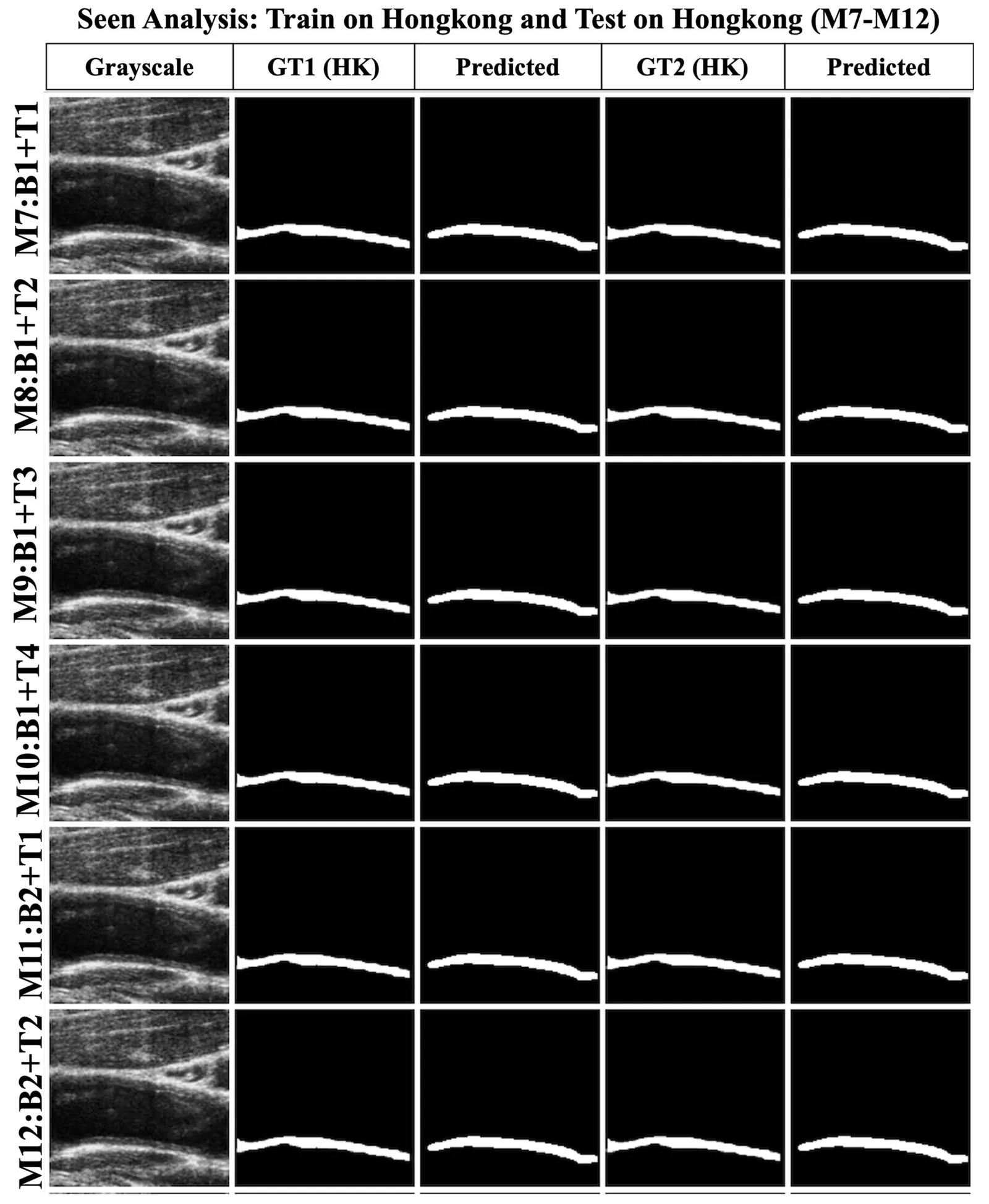
Seen-domain segmentation performance where the model is trained and evaluated on the Hong Kong dataset (M7–M12).

Figure 7 illustrates qualitative results for the seen-domain analysis, in which the model is trained and tested on the HongKong dataset. The figure shows grayscale inputs, corresponding ground-truth masks (GT1 and GT2), and predicted segmentation outputs for Models 7–12.

**Figure 8:**
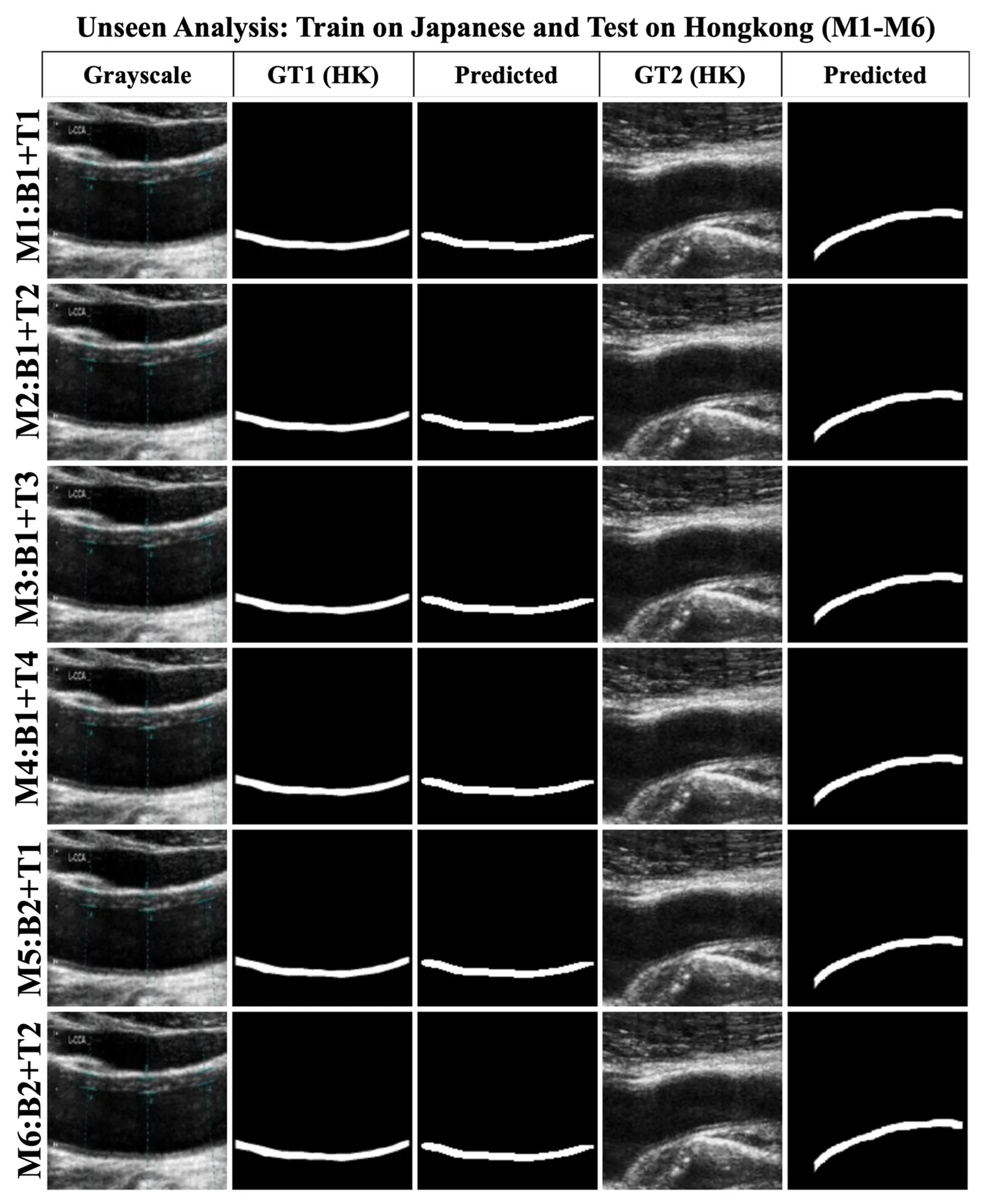
Unseen-domain segmentation performance where the model is trained on Japanese and evaluated on the Hong Kong dataset (M1–M6).

Figure 8 illustrates the unseen analysis, in which the model is trained on the Japanese dataset and tested on the Hong Kong dataset. The figure shows grayscale inputs, corresponding ground-truth masks (GT1 and GT2), and predicted segmentation outputs for Models.

**Figure 9:**
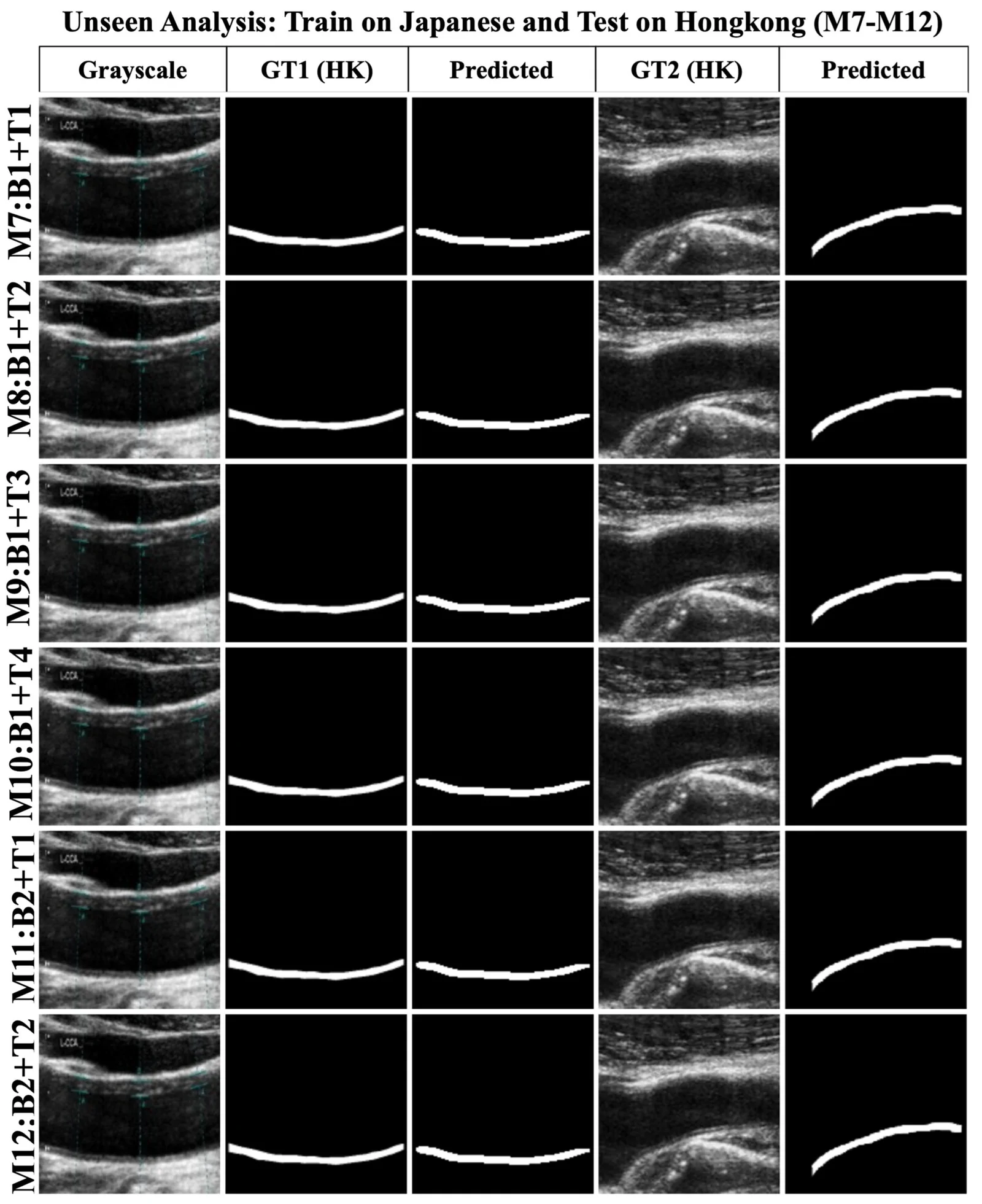
Unseen-domain segmentation performance where the model is trained on Japanese and evaluated on the Hong Kong dataset (M7–M12).

Figure 9 illustrates the unseen-domain analysis, in which the model is trained on the Japanese dataset and tested on the HongKong dataset. The figure shows grayscale inputs, corresponding ground-truth masks (GT1 and GT2), and predicted segmentation outputs for Models.

## 5. Performance Evaluation

### 5.1. Percentage Improvement

**Figure 10:**
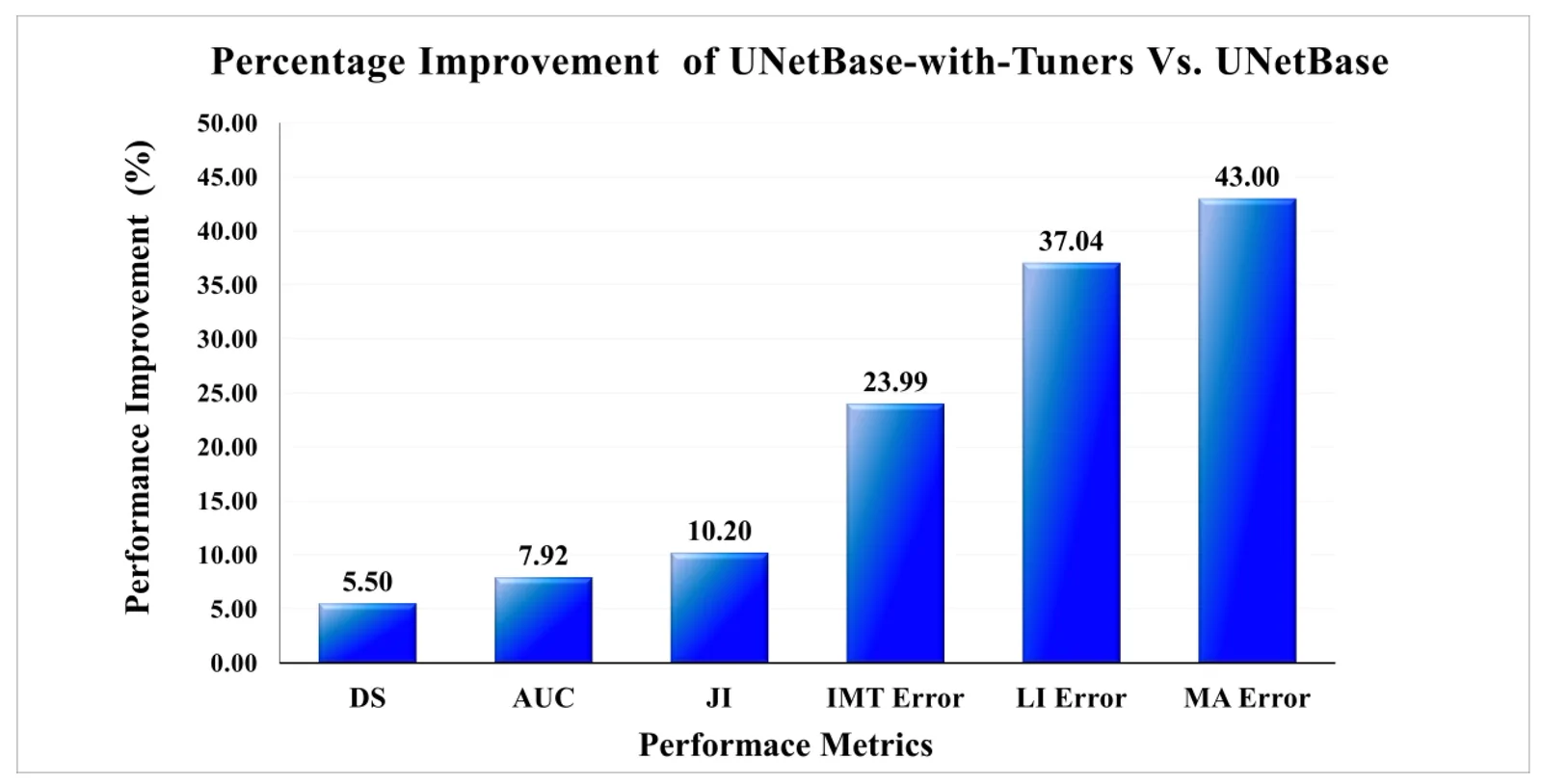
Percentage improvement of UNet-base tuners over UNet-base.

Figure 10 presents the percentage mean performance gains of the proposed UNetBase-with-Tuner over the baseline UNetBase. Significant improvements are observed in MA Error (43.08%), LIE (37.04%), and IMT Error (23.99%).

### 5.2. Area Under the Curve

**Figure 11:**
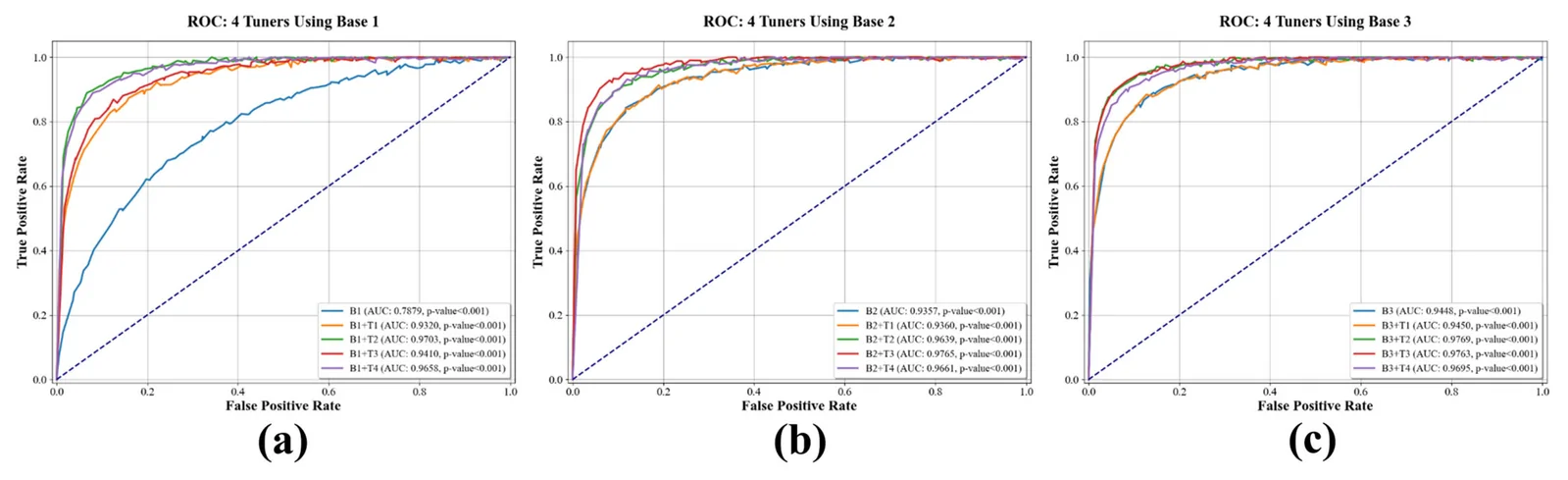
(**a**) ROC Curves using B1 with all Tuners (**b**) ROC Curves using B2 with all Tuners, and (**c**) ROC Curves using B3 with all Tuners.

Figure 11 demonstrates that integrating tuners (T1–T4) with the base models (B1–B3) consistently improves AUC performance across all configurations. The best AUC was obtained for B3 + T4, which demonstrates its best segmentation power in the carotid wall.

### 5.3. Memorization vs. Generalization Analysis

**Figure 12:**
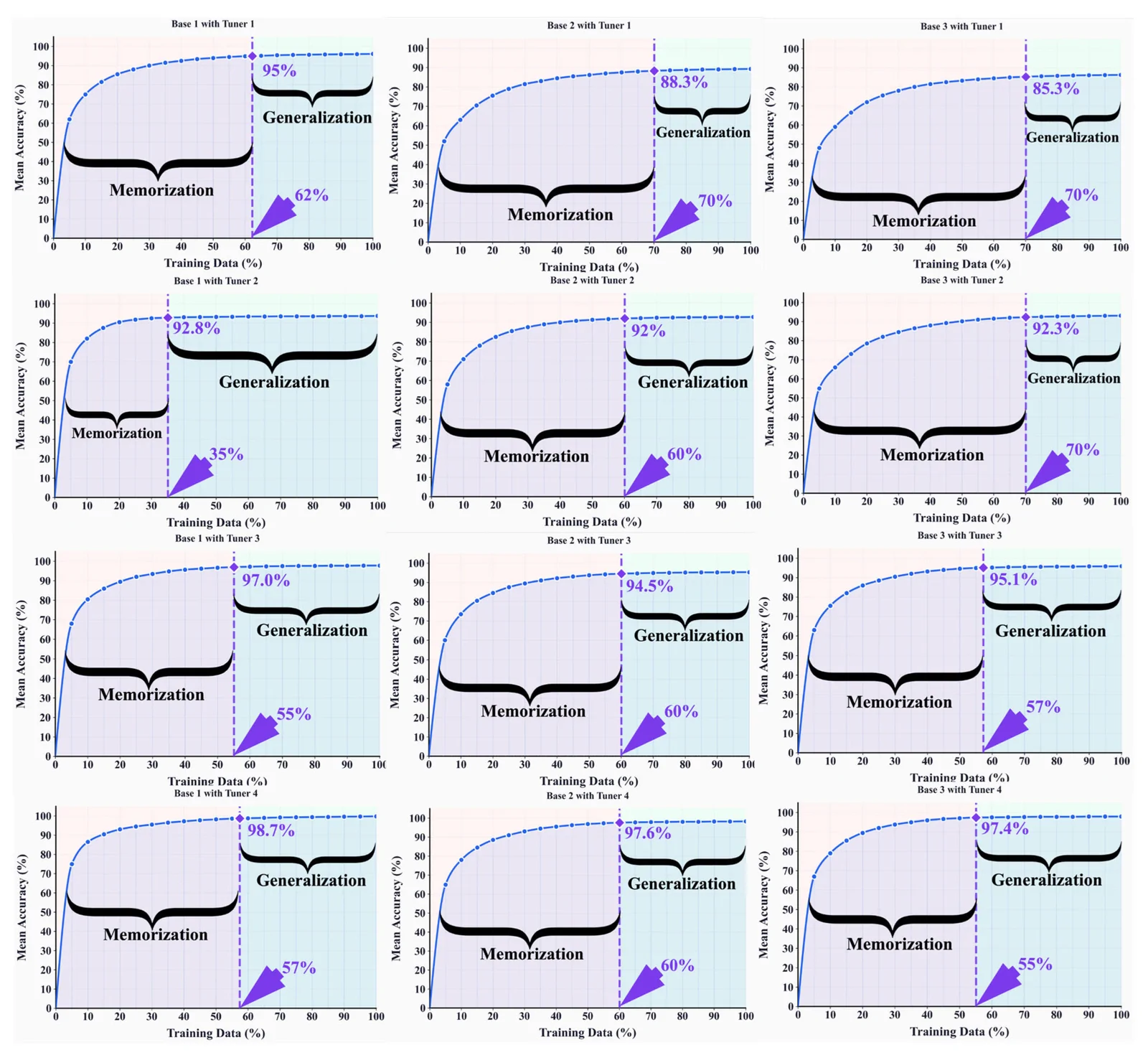
Generalization vs. Memorization of 12 Models (M1–M12).

Figure 12 analyzes the balance between memorization and generalization across the proposed tuner-enhanced models. The results highlight the models’ ability to reduce overfitting while achieving strong generalization performance with increasing training data.

## 6. Scientific Validation and Software Verification

### 6.1. Effect of Augmentation on Base Models (Stage I)

The base models were first evaluated in isolation to establish reference performance. As reported in Table 1, each base network was trained and assessed both with and without data augmentation, thereby isolating the marginal contribution of augmentation to segmentation accuracy and robustness.

### 6.2. Impact of Augmentation on the Best Fused System

Table 2 represents the experiments conducted on models with and without data augmentation techniques to gauge their performance.

### 6.3. K-Parameter Sensitivity

We studied the effect of increasing and decreasing the value of K (e.g., K-fold cross-validation or in layers of a model dependent) on the overall performance of the model. Table 3 shows how the performance of the Base 3 + Tuner 4 model varies on changing K factor.

Train-Test (TT): Configuration in which Full of the data set was used for training and the other Full for testing, to get a theoretical upper-bound estimate of the capacity of the model rather than the generalization performance. KM5 (K-Minus-5): Reversed the splitting with 20% data used for training and 80% data used for testing, assessing the robustness of the model under limited training data. The columns reported are not for main performance claims, and all of them are based on K-fold cross-validation (K2–K10).

### 6.4. Enhancement by Tuner Stage 

A systematic hyperparameter optimization stage was subsequently introduced. As summarized in Table 4, adjustment of the learning rate, batch size, dropout rate, and optimiser configuration yielded consistent improvements in segmentation accuracy, convergence stability, and cross-cohort generalization.

### 6.5. Unseen Analysis

Complementing the qualitative panels in Figure 8 and Figure 9, all twelve fused models were trained on DB1 (Japanese) and evaluated without retraining on the independent DB2 (Hong Kong) cohort, which differs in scanner, ethnicity, and clinical profile. Table 5 reports the resulting seen and unseen-domain performance across DS, JI, and cIMTe. Across all configurations the unseen-domain degradation remained narrow, with F12 (B3 + T4) retaining the best performance at 91.05% DS and a cIMTe of 0.021 mm.

### 6.6. External Validation

To evaluate the framework under a true unseen-domain setting, we performed external validation on the Carotid Ultrasound Boundary Study (CUBS) database, an open, CC BY 4.0-licenced multi-centre resource released by Meiburger et al. comprising longitudinal B-mode common carotid scans from 1088 patients across two independent European centres, with both sides of the neck imaged (2176 scans; case identifiers of the form Clin_0266_L, Clin_0729_R denote the clinical case and imaged side). CUBS is disjoint from our training cohorts (DB1, Japanese; DB2, Hong Kong post-menopausal).

**Figure 13:**
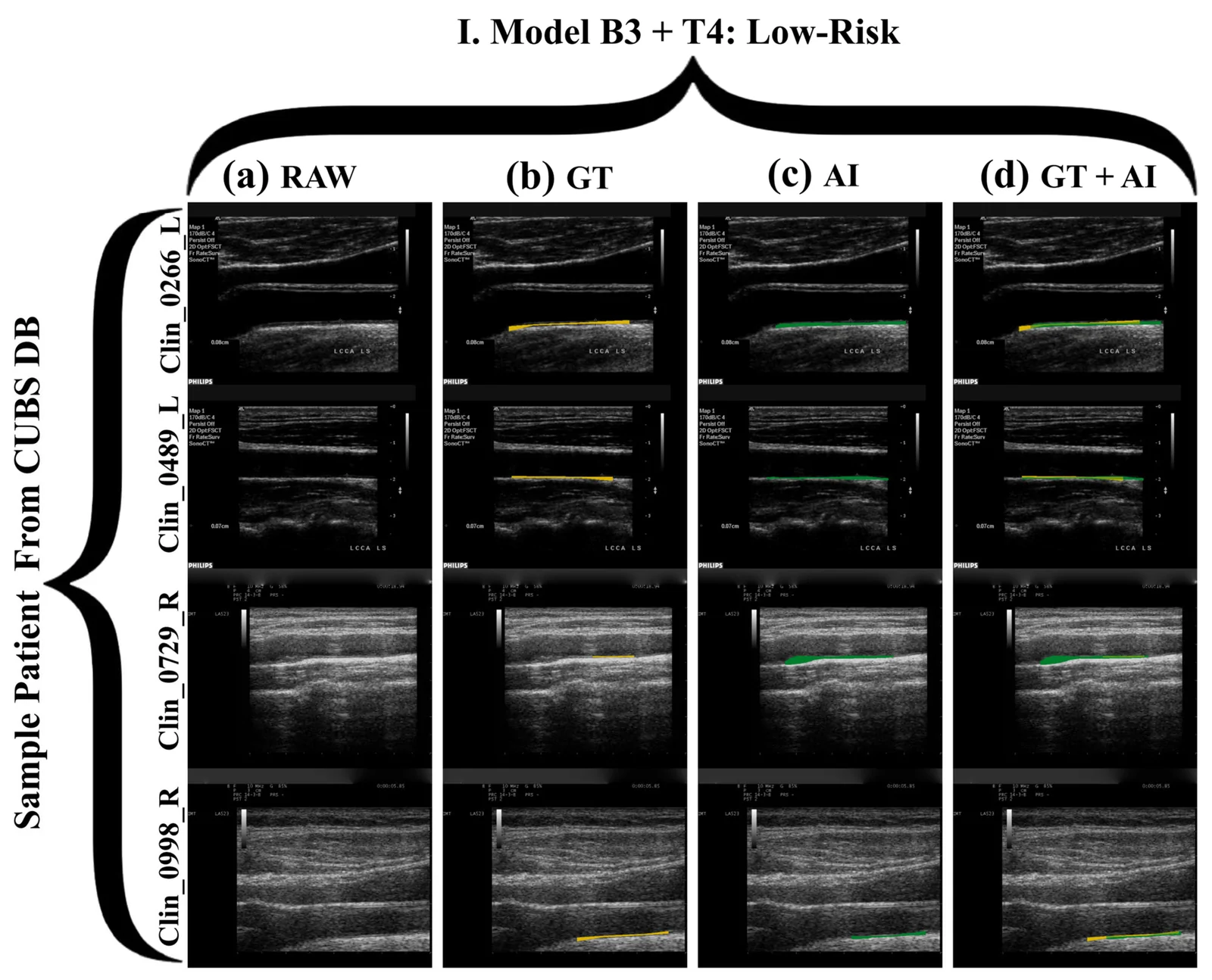
Model B3 + T4 on low–risk CUBS DB patients: (**a**) RAW, (**b**) GT, (**c**) AI, (**d**) GT + AI.

Figure 13 presents four low-risk CUBS patients. (a) Represents the original grayscale image, and the AI tracings (column c) overlap the expert ground-truth (column b) almost throughout.

**Figure 14:**
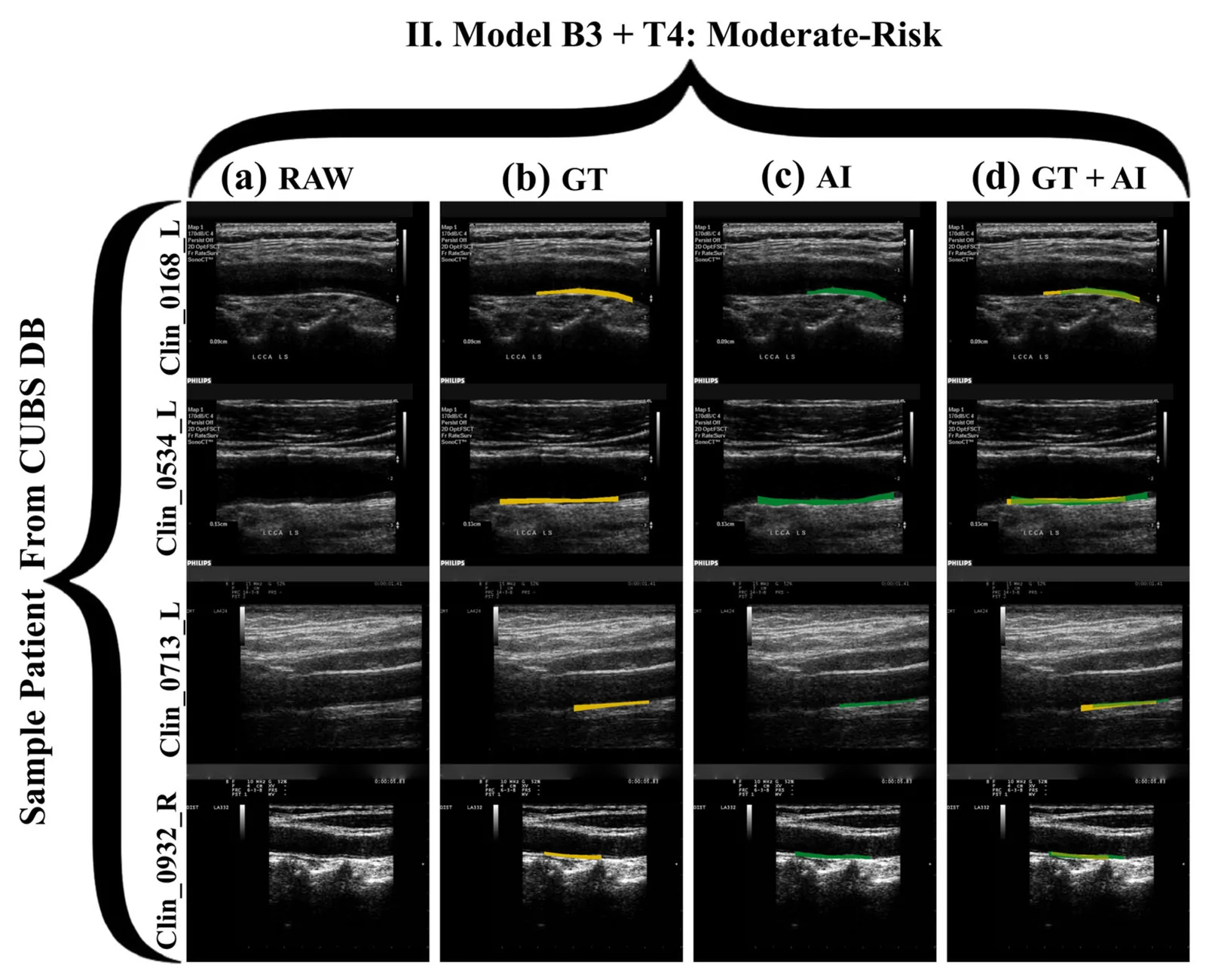
Model B3 + T4 on moderate–risk CUBS DB patients: (**a**) RAW, (**b**) GT, (**c**) AI, (**d**) GT + AI.

Figure 14 shows four moderate-risk CUBS patients (Clin_0166_L, Clin_0534_L, Clin_0713_L, Clin_0932_R), in which increased wall thickening and greater speckle contamination raise the segmentation difficulty. The model continues to recover both boundaries with the expected wall geometry, with the GT + AI overlay showing only localized departures from expert tracing.

**Figure 15:**
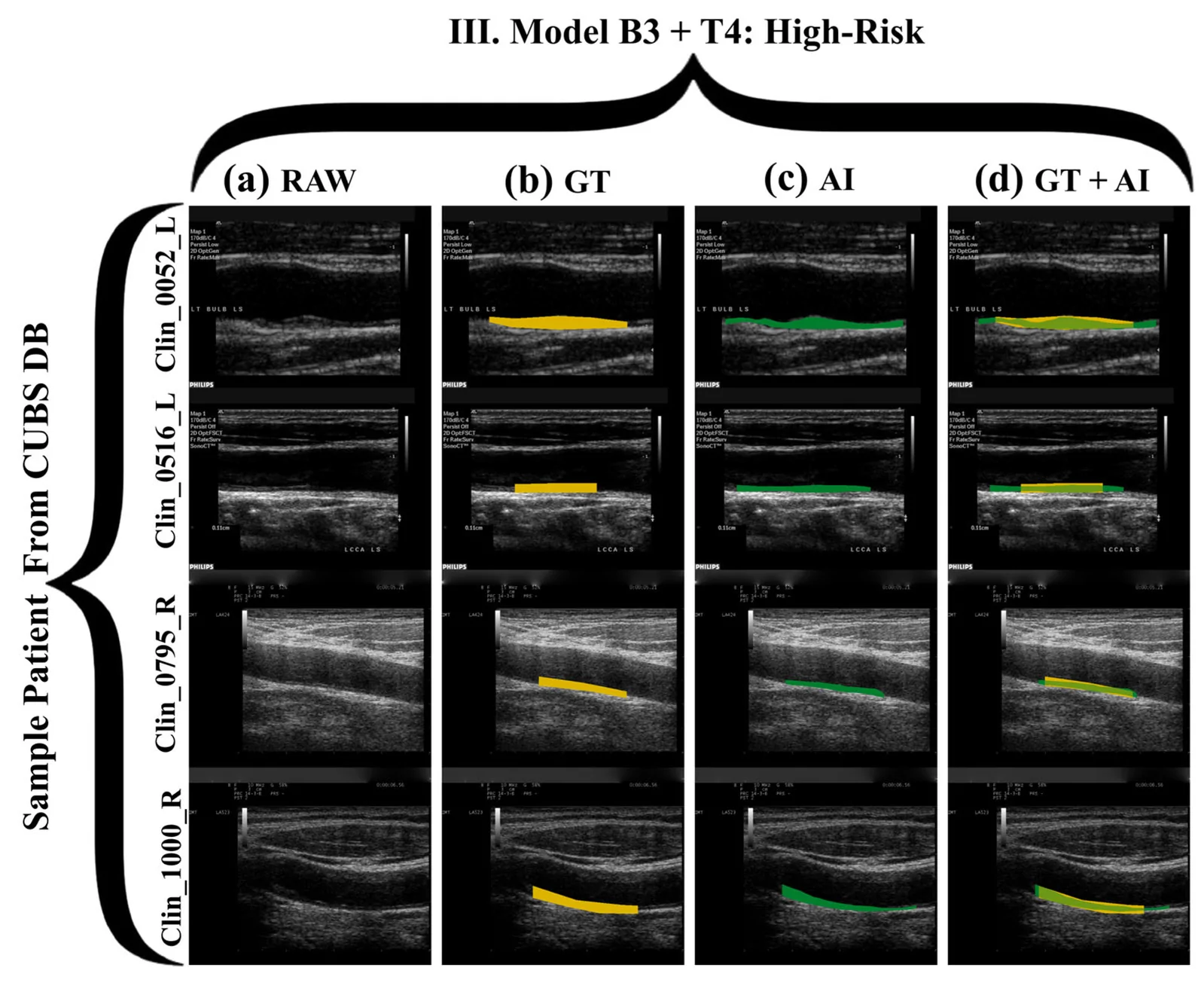
Model B3 + T4 on high–risk CUBS DB patients: (**a**) RAW, (**b**) GT, (**c**) AI, (**d**) GT + AI.

Figure 15 reports four high-risk CUBS patients (Clin_0852_L, Clin_0546_L, Clin_0795_R, Clin_1000_R), characterized by pronounced wall thickening and irregular boundary morphology. Despite these conditions, the AI-predicted LI and MA contours remain continuous and anatomically plausible, with the overlay indicating retained agreement with the expert reference in the most challenging stratum of the unseen cohort.

**Figure 16:**
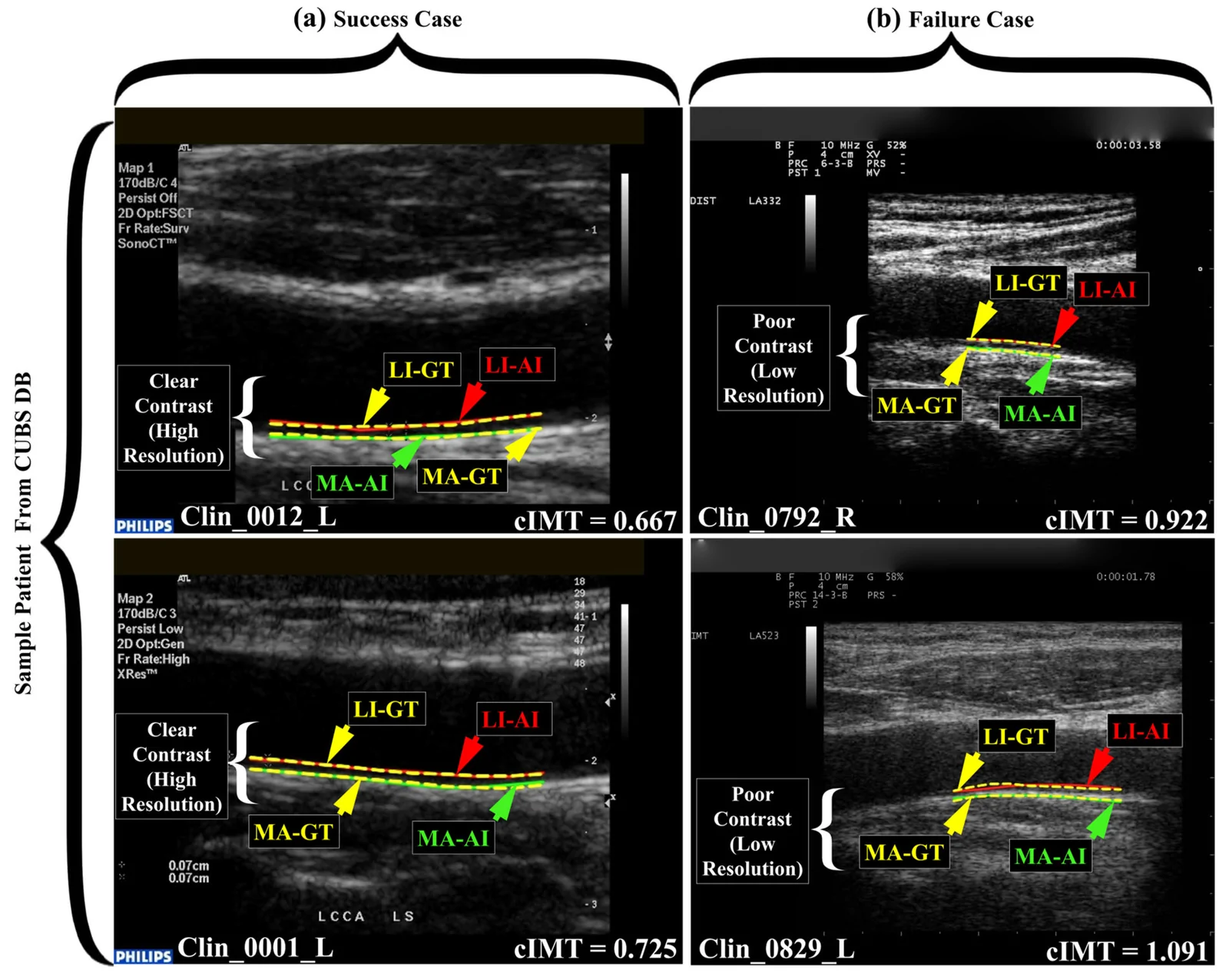
Success and failure cases of Model B3 + T4 on CUBS DB: (**a**) success (**b**) failure cases LI/MA–GT (yellow) vs. LI/MA-AI (red/green), with corresponding cIMT values (mm).

To characterize the conditions under which the framework succeeds or degrades on the unseen cohort, Figure 16 contrasts representative success and failure cases from CUBS DB for the strongest fused configuration (Model B3 + T4). In the success cases (Figure 16a; Clin_0012_L, Clin_0001_L), the intima–media complex is imaged at high resolution with clear acoustic contrast at both interfaces, and the AI-predicted lumen–intima (LI-AI) and media–adventitia (MA-AI) contours coincide almost exactly with the expert tracings (LI-GT, MA-GT), yielding cIMT values of 0.667 mm and 0.725 mm respectively. In the failure cases (Figure 16b; Clin_0792_R, Clin_0829_L), low spatial resolution and poor contrast blur the LI and MA interfaces, causing the predicted boundaries to drift from the reference and inflating cIMT to 0.922 mm and 1.091 mm. This pattern indicates that residual error on the unseen domain is driven predominantly by acquisition quality rather than by wall morphology and identifies image contrast and resolution as the principal limiting factor for cross-cohort deployment.

## 7. Comparative Analysis

**Figure 17:**
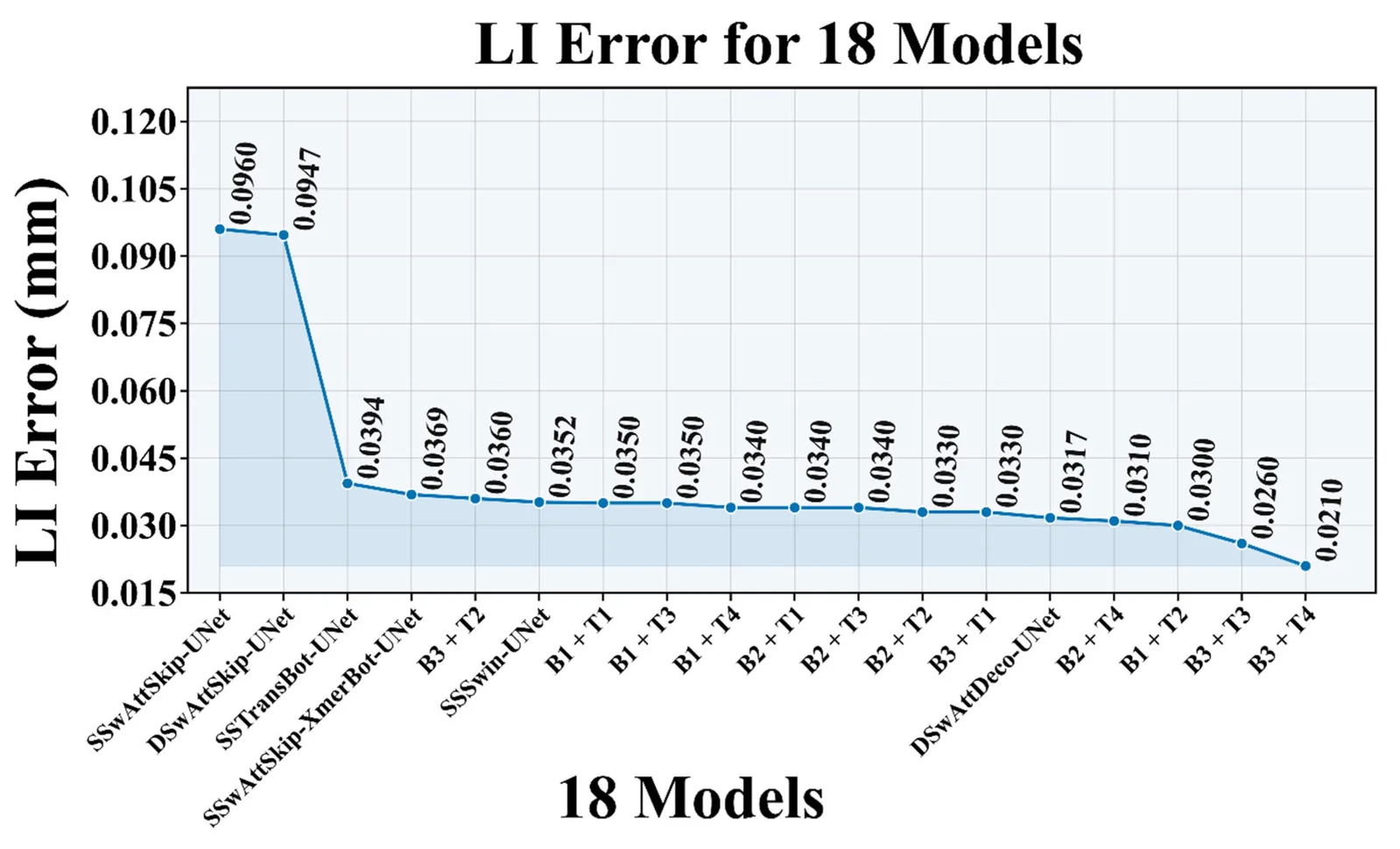
Lumen–Intima Error across 18 models, ranked in descending order.

In this section, we compare state-of-the-art models on the different clinical metrics. Figure 17 ranks all 18 evaluated configurations, the six standalone base networks, and the twelve fused base + tuner combinations by lumen–intima (LI) boundary error on the seen Japanese cohort. The fused configurations occupy the low-error tail of the ranking, with Model B3 + T4 attaining the minimum LI error of 0.0210 mm, confirming that the transformer-tuner stage yields its largest gains precisely at the interface most affected by lumen speckle.

**Figure 18:**
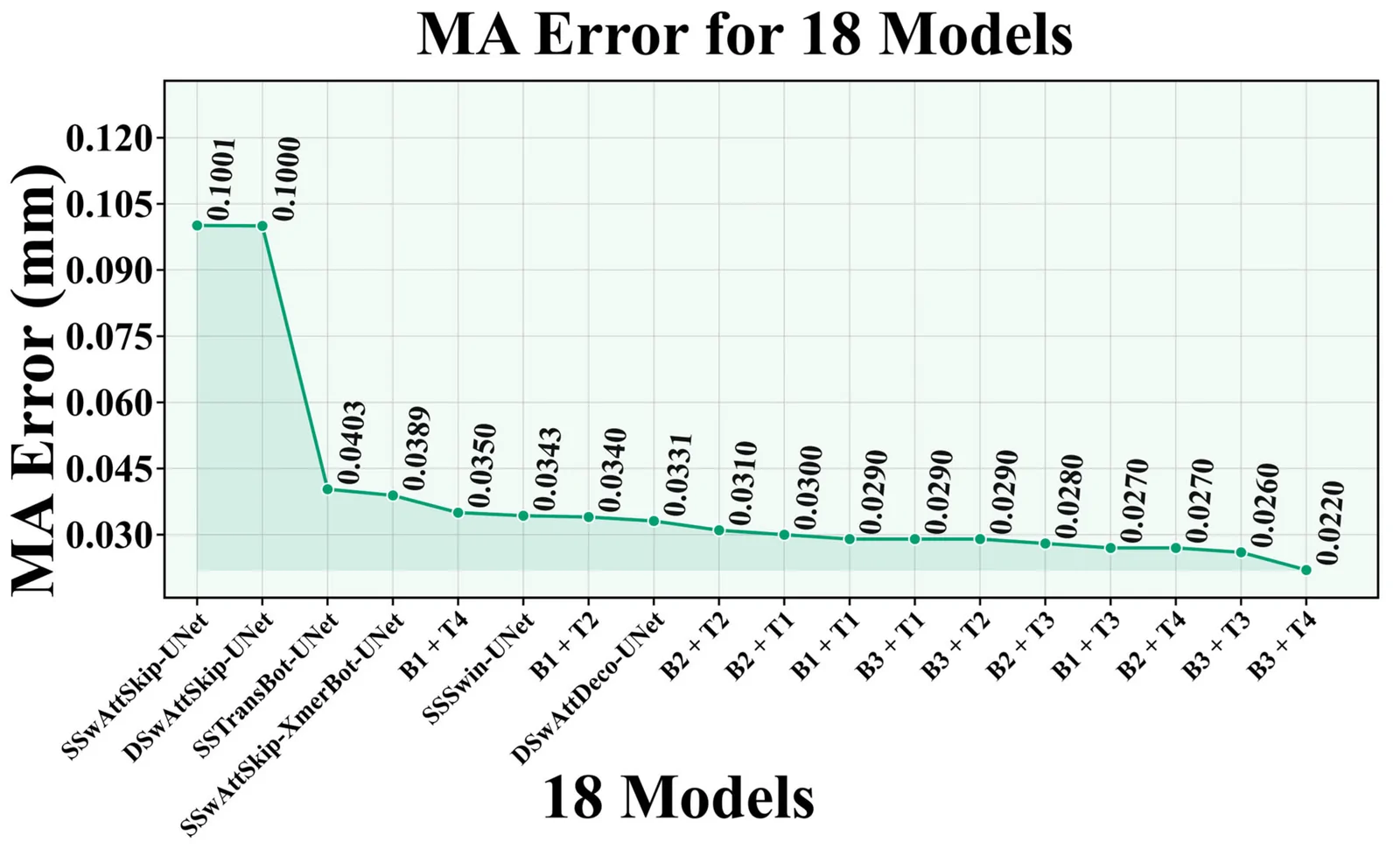
Media-Adentitia Error across 18 models, ranked in descending order.

Also, Figure 18 reports the corresponding media–adventitia (MA) boundary error for all 18 evaluated configurations.

**Figure 19:**
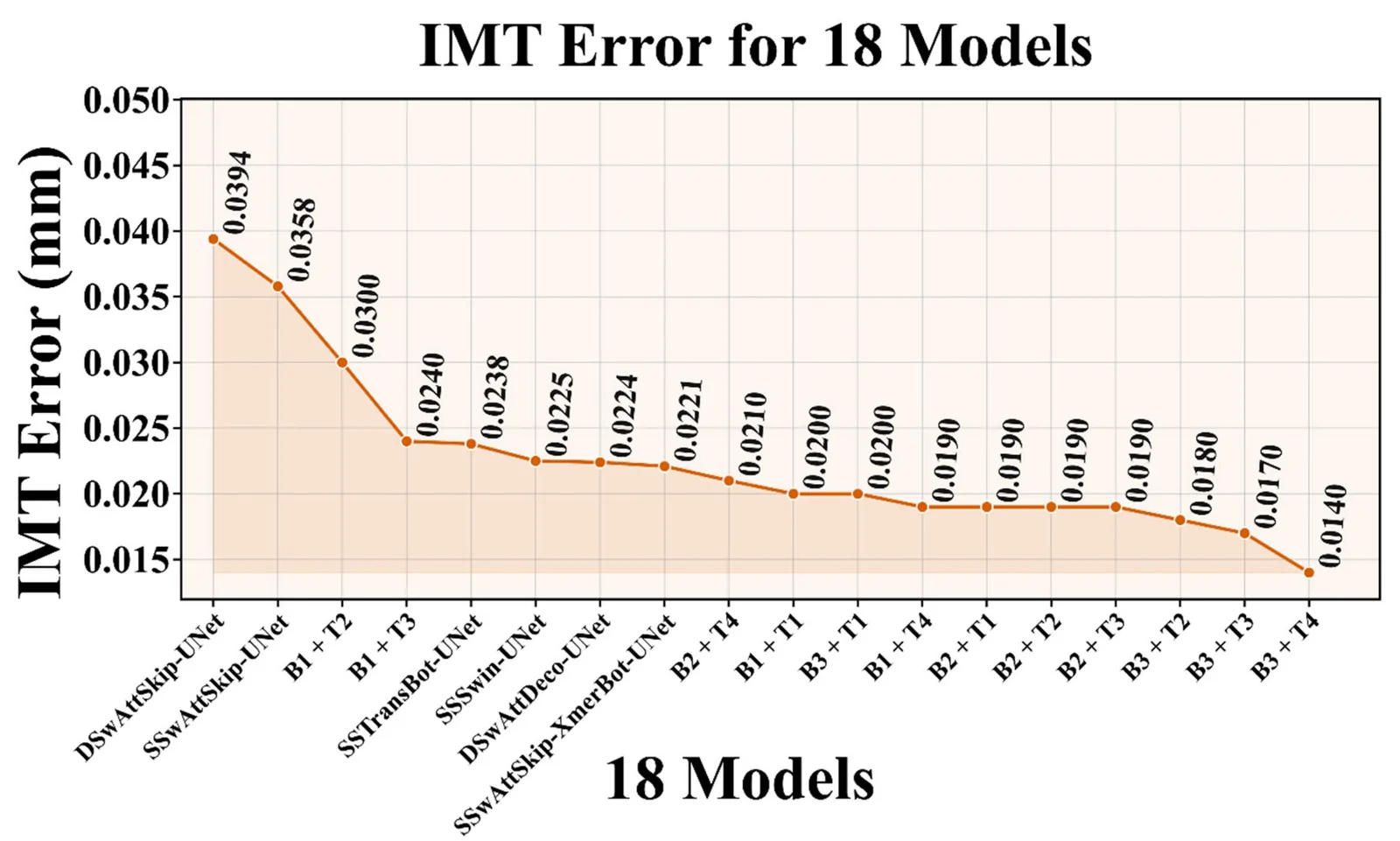
Intima-Media Thickness Error across 18 models, ranked in descending order.

Similarly, Figure 19 reports the corresponding Intima-media Thickness (IMT) boundary error for the same 18 configurations on the Japanese database.

**Figure 20:**
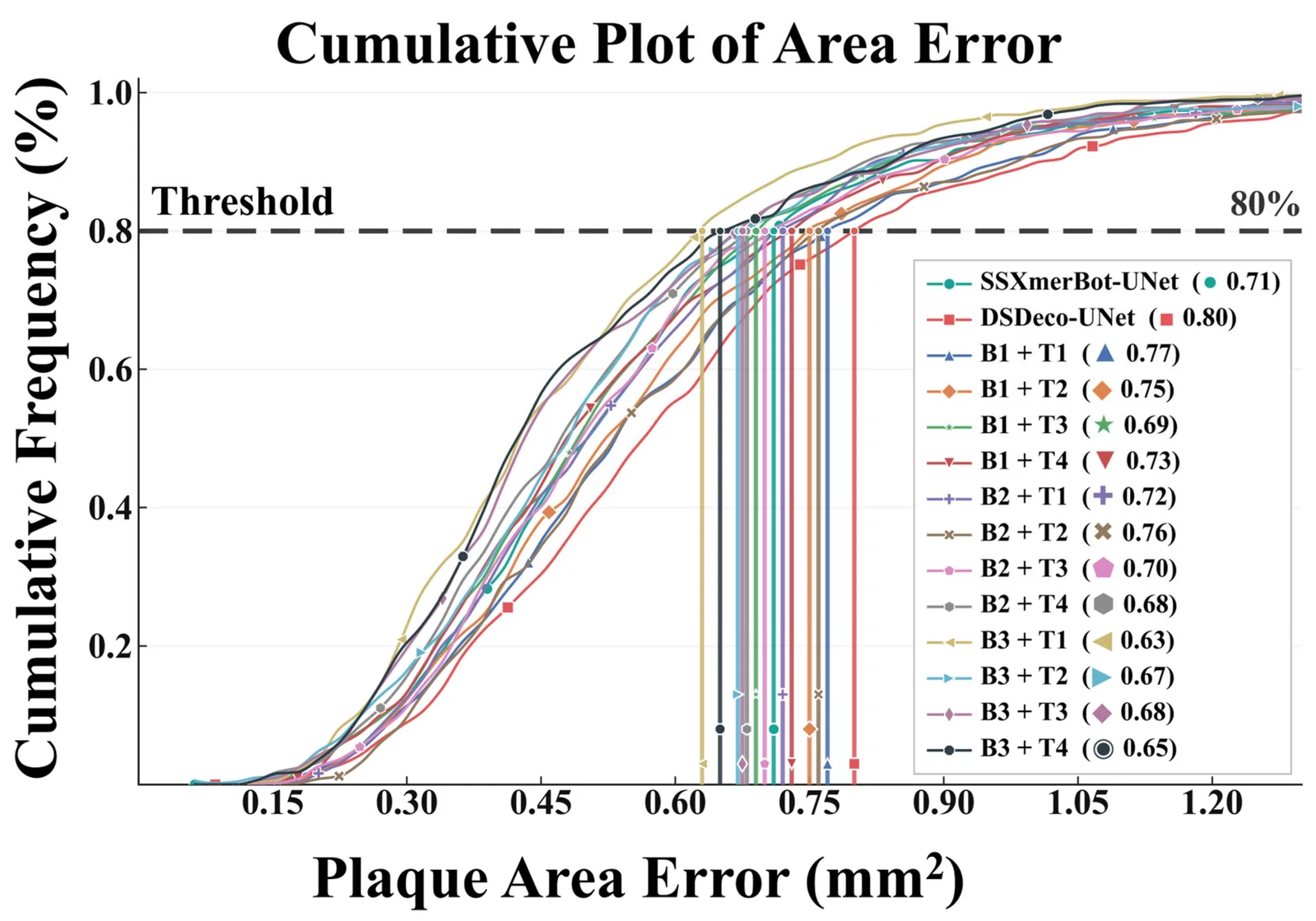
Cumulative Plot of Area Error for various methods.

Now, Figure 20 reports the corresponding cumulative plot of Total Plaque Area Error (TPAe) for all 18 evaluated configurations.

**Figure 21:**
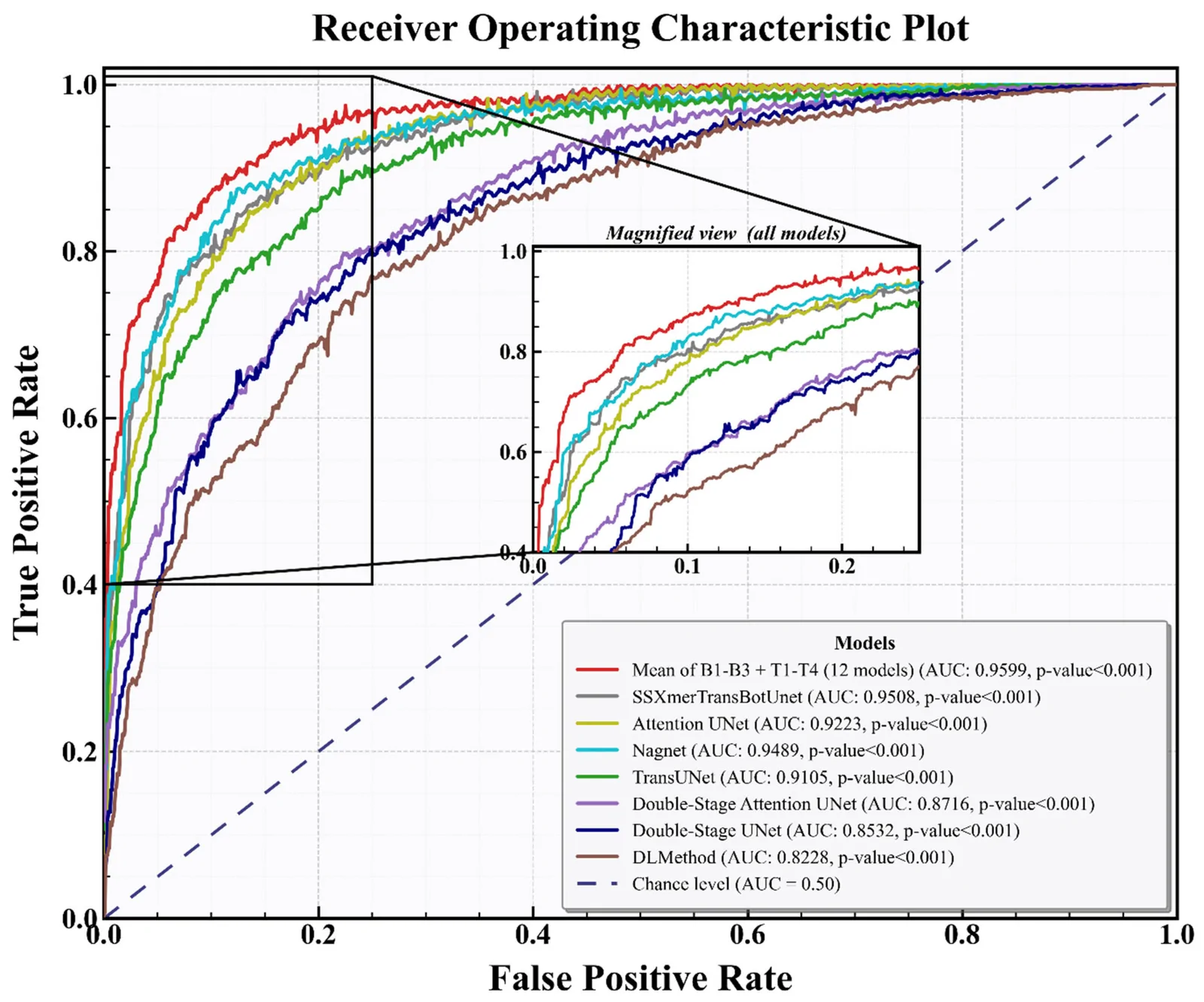
Receiver Operating Characteristics for various methods.

Figure 21 shows the receiver operating characteristic (ROC) curves for the proposed twelve-model fused framework (mean of B1–B3 + T1–T4) and seven benchmark methods. The fused framework achieved the highest area under the curve (AUC = 0.9599), followed by SSXmerTransBotUnet (0.9508) and NAG-Net (0.9489), with all remaining models ranging from 0.9223 down to 0.8228 (DLMethod). Every AUC was statistically significant (*p* < 0.001) and well above the chance diagonal (AUC = 0.50). The inset magnifies the low-false-positive-rate region, where the fused framework consistently attained the greatest true positive rate indicating high sensitivity at low false-alarm rates for carotid wall delineation.

Table 6 presents a detailed comparison between the deep learning architectures (M1–M8) used in this study. The models evaluated are as follows: (i) Model M1: UNet, which serves as the baseline with a simple architecture based on the original UNet concept [44]; (ii) Model M2: DL Method (iii) Model M3: Attention-UNet, which adds attention mechanisms to skip connections to enhance spatial feature extraction and robustness [45]; (iv) Model M4: NAG-Net, designed by stacking two attention-based single-stage UNets to compare single- versus multi-stage performance; (v) Model M5: A *Single-Stage Transformer-based UNet (TransUNet)* consists of transformer embedded in bottleneck layer [32], (vi) Model M6: A *Single-Stage Swin-Transformer-based UNet (SwinUNet)* consists of transformer embedded in every layer (vii) Model M7: A *Single-Stage Transformer-bottleneck UNet (SSwAttSkip-XmerBot-UNet model)* which integrates transformer blocks to and attention in skip for improved contextual understanding. (viii) Model M8: B3 + T4, Our best proposed and base–tuner combination model. To assess model architectures (M1–M8) in Table 6, we arrange them into three buffers.

The first buffer, which is the performance, indicates the quality of segmentation and predictability. In this buffer, metrics used were: (1) Dice Score (DS) and Jaccard Index (JI), the measures of overlap between predicted and ground-truth areas, (2) Bland–Altman (BA) plot, which measures the correlation between predicted and measured areas.

The second buffer is efficiency, which identifies the requirements of computers and memory. The measurements made were: (a) Inference Latency (ms) which is calculated as the mean forward-pass time, Training Time (TrgTi) which is the total time to converge (b) Number of Parameters (P#), (c) Energy Usage, (d) Storage Size (S#) which is the model disc size, and (e) Memory Usage (Mb), which is the maximum use of RAM Such low numbers indicate quicker, lighter, and more movable models.

The third buffer is measurements, measures the accuracy of predictions in comparison to the ground truth with clinically significant readings. Measures were: (a) cIMTE (Intima-Media Thickness Error), calculated as the difference between predicted and true Intima-Media Thickness to assess the accuracy of arterial wall segmentation; (b) LIE (Lumen–Intima Error), the difference between the predictions of the lumen–intima boundary and the actual ones, to measure the accuracy of lumen segmentation; (c) MAE (Media–Adventitia Error), the difference between the predicted and actual means of the media–adventitia boundary, which is a measure of the accuracy of the outer vessel wall; (d) PAE Lower values of these metrics denote closer agreement with the reference tracings; among the benchmarked architectures, M4 achieved the smallest LIE, MAE, and cIMTE, and was surpassed only by the proposed configuration M8 (B3 + T4). Table 6 summarizes the evaluation of architectures M1–M8, organized into three metric groups (performance, efficiency, and measurement accuracy) to provide a multidimensional basis for model selection in clinical deployment.

## 8. Variability Analysis

This study follows a supervised semantic segmentation framework requiring pixel-level annotations for lumen–intima (LI) and media–adventitia (MA) boundary detection. The arterial interfaces and the plaque regions were manually outlined by experienced sonographers and cardiologists and then independently verified to ensure annotations consistency. The annotated contours were converted into binary masks using a MATLAB R2014a-based pipeline, and all grayscale images and masks were resized to 256 × 256 pixels for model training. Here, GT1 denotes the Japanese cohort, GT2 the Hong Kong cohort, and GT3 the same Japanese cohort as GT1 annotated at a different time by a different operator. Inter-observer variability was assessed through regression and Bland–Altman analyses, demonstrating strong agreement among GT1, GT2, and GT3 annotations. Annotation variability was observed within the 1–5% range.

### 8.1. Regression Plots

**Figure 22:**
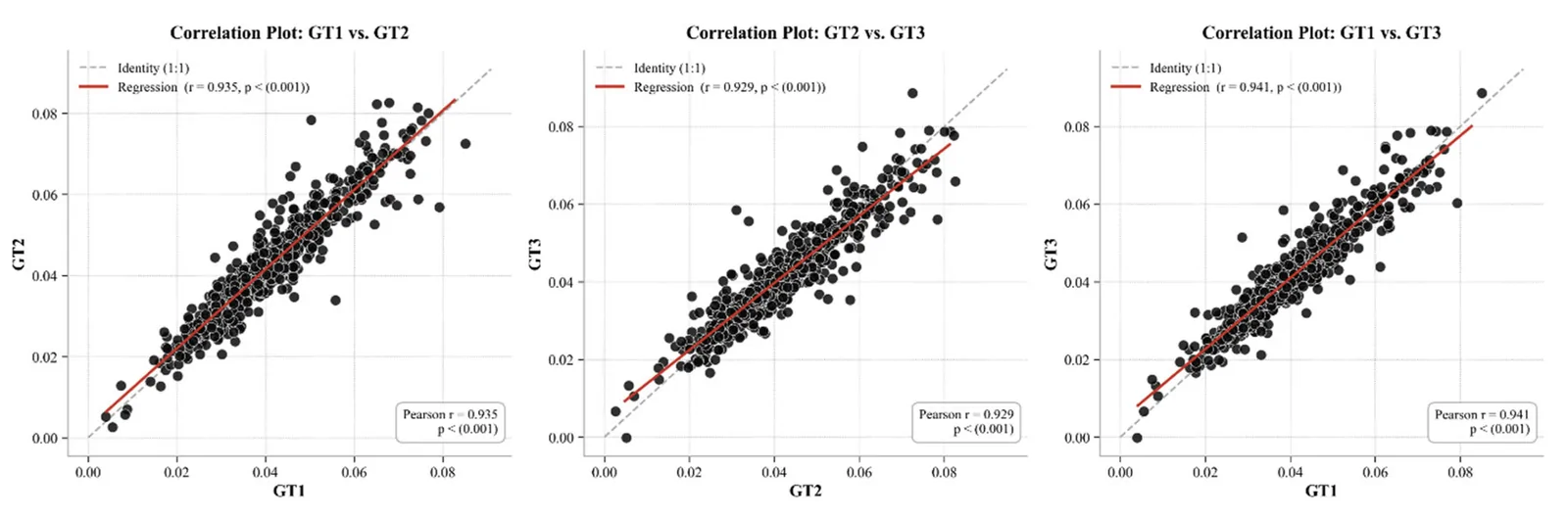
Correlation Plots using GT1, GT2 and GT3.

Figure 22 presents the regression analysis performed between different ground-truth annotations. The correlation plots demonstrate a strong linear relationship and high agreement among GT1, GT2, and GT3 measurements.

### 8.2. Bland-Altmann’s Plot

**Figure 23:**
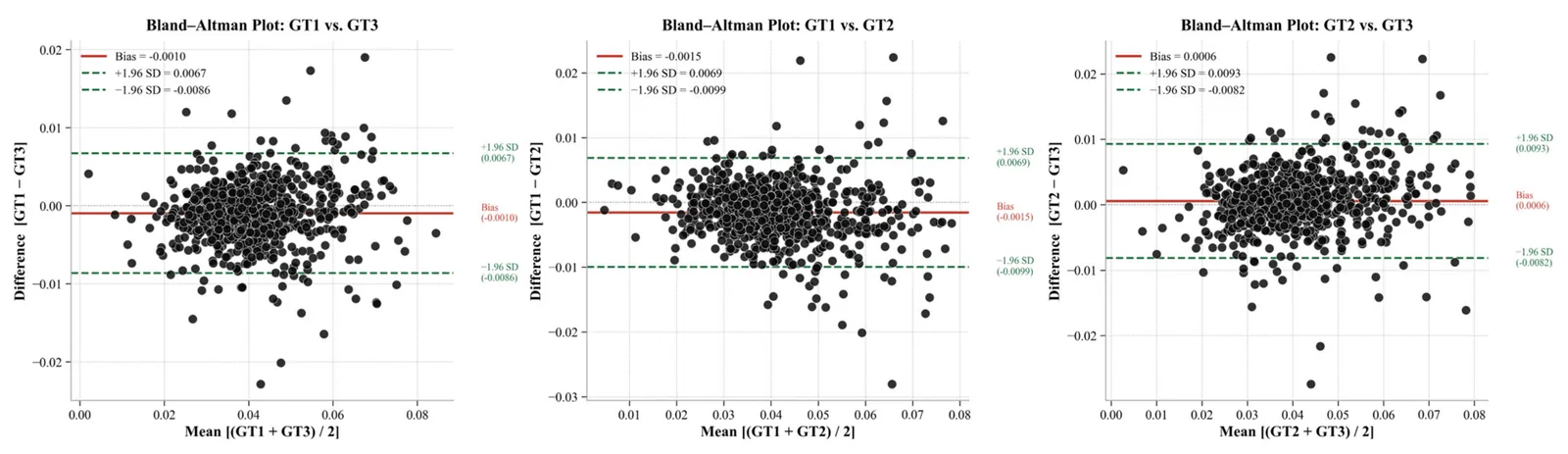
Bland–Altmann Plots using GT1, GT2 and GT3.

Figure 23: The Bland–Altman analysis [46] for agreement assessment between different ground-truth annotations. The plots demonstrate consistency and reliability.

## 9. Statistical Tests

Table 7 presents the pairwise statistical comparison of model performances using the *t*-test, Mann–Whitney U Test, and Wilcoxon Test.

Most pairwise comparisons were achieved statistical significance (*p* < 0.05) across all three tests. Borderline non-significant values observed for Base 1 + Tuner 1 vs. Base 1 + Tuner 2 (Mann–Whitney *p* < 0.065, Wilcoxon *p* < 0.055) and Base 2 + Tuner 1 vs. Base 3 + Tuner 1 (Wilcoxon *p* < 0.055) are attributable to the narrow performance margins between configurations already operating at high accuracy levels, with corresponding *t*-test results remaining significant (*p* < 0.01).

## 10. Explainability

**Figure 24:**
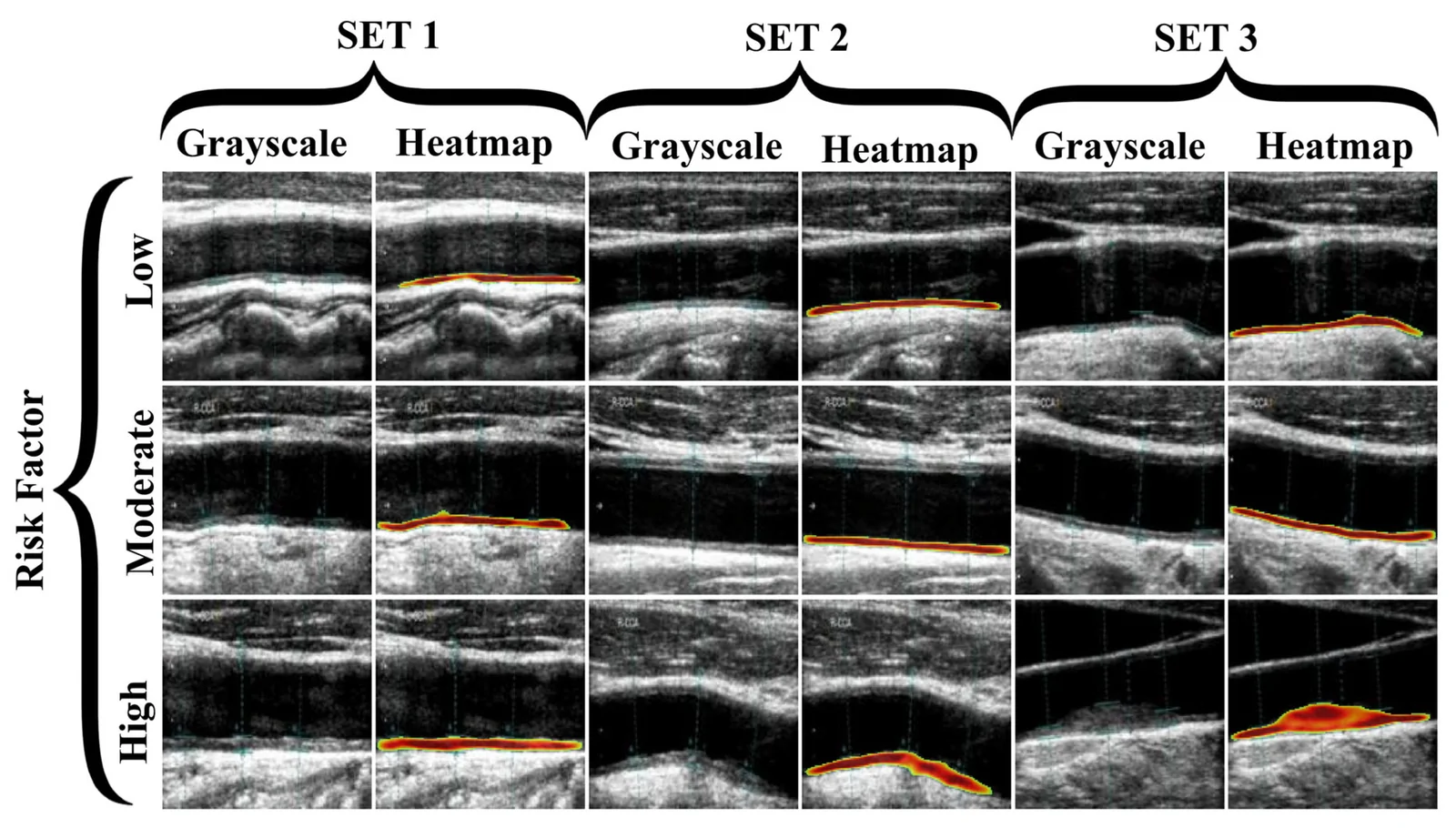
Heatmaps using Japanese database.

**Figure 25:**
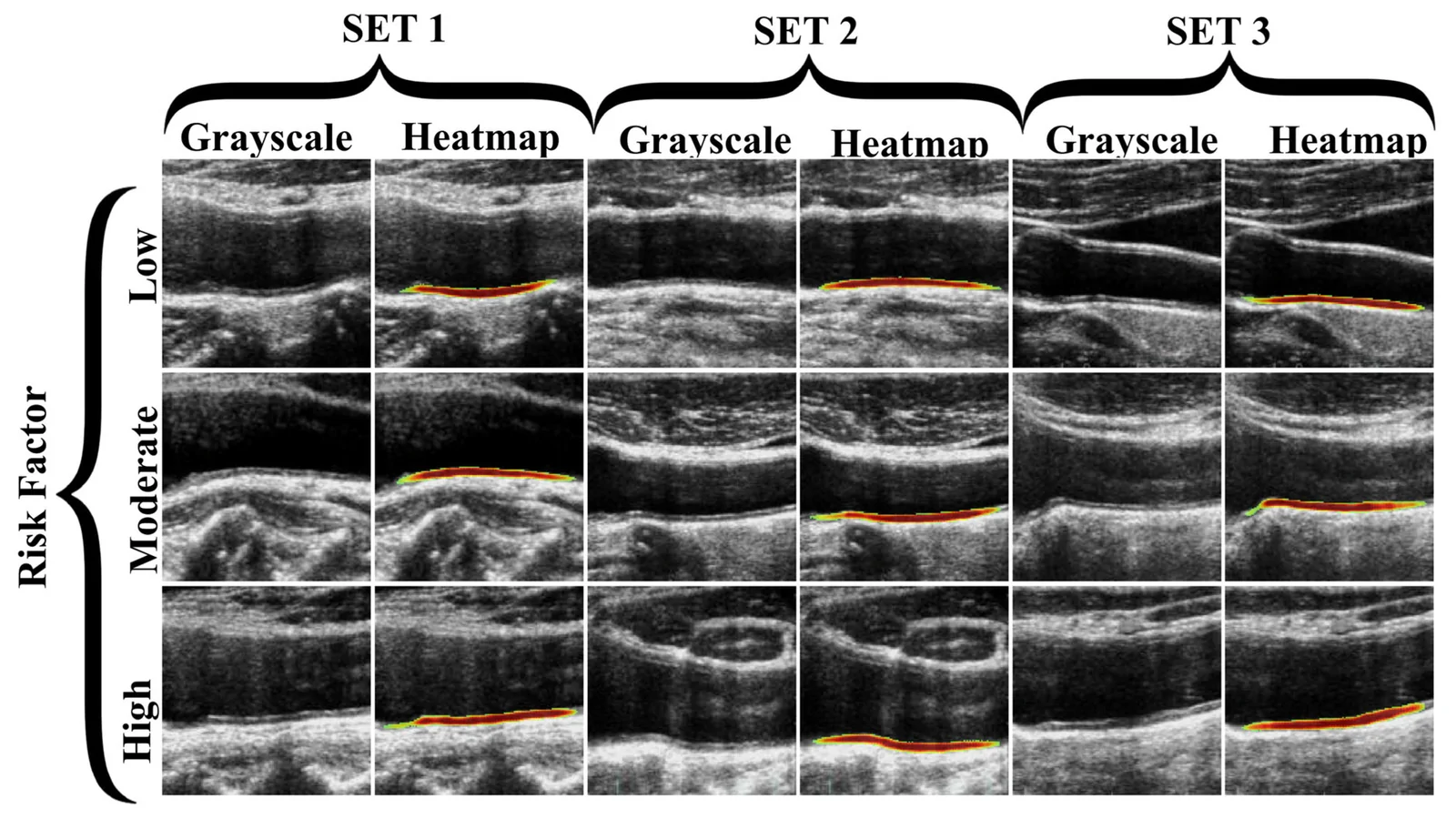
Heatmaps using Hong Kong database.

Interpretability is a prerequisite for the clinical adoption of automated diagnostic systems, since accuracy alone provides insufficient grounds for clinical trust. In carotid analysis, transparent attribution is required to establish that automated risk estimates derive from anatomically meaningful vascular features rather than from incidental image characteristics. In carotid analysis, transparency establishes that automated risk decisions rest on meaningful vascular characteristics rather than incidental image patterns. We therefore examine the decision-making behaviour of the proposed models using Layer-wise Relevance Propagation (LRP) [47], which attributes the network output back to the input by propagating a relevance signal through successive layers under a conservation constraint, yielding a pixel-level attribution map. These maps are fused with the self-attention distributions of the transformer-tuner stage and overlaid on the grayscale scan. Results are reported for three model sets across the low- (cIMT < 0.4 mm), moderate- (0.4 ≤ cIMT ≤ 0.8 mm), and high-risk (cIMT > 0.8 mm) strata of both training cohorts: the Japanese database (Figure 24) and the Hong Kong post-menopausal database (Figure 25).

Both cohorts show a coherent progression across risk strata. In low-risk cases, relevance is smooth and evenly distributed along the vessel wall, consistent with uniform, healthy carotid boundaries. In moderate-risk cases, it intensifies around localized thickening, indicating sensitivity to early atherosclerotic structures. In high-risk cases, activations concentrate over plaque-dense and morphologically irregular regions. The stability of this pattern across two cohorts differing in scanner, ethnicity, and clinical profile confirms that the model’s focus tracks clinical severity rather than cohort-specific artefacts, allowing its diagnostic reasoning to be both mathematically traced and visually verified.

## 11. Critical Discussion

The principal methodological advantage of the proposed two-stage framework lies in the separation of local and global optimization, which permits spatial refinement and contextual refinement to be pursued independently. Stage I employs base UNet variants (B1–B3) to trace boundaries hierarchically across scales, thereby preserving fine anatomical detail and ensuring robust edge localisation in the presence of speckle and low interface contrast [39]. Stage II introduces global context through transformer-based tuners and attention-guided refinement, which is of particular benefit in low-contrast and morphologically heterogeneous regions [40,48]. Across the twelve evaluation criteria applied in this study, the cascaded design consistently exceeded the corresponding single-stage configurations in accuracy and cross-cohort stability, while maintaining an acceptable accuracy-to-parameter ratio; the consistency of these gains under K-fold cross-validation and augmentation ablation supports their attribution to the architecture rather than to a particular training configuration.

However, there are also practical issues of memory utilization, inference speed, and component transformer interpretability [36,49]. Unlike existing single-stage hybrid approaches such as TransUNet and Swin-UNet, which embed transformer modules directly within the encoder–decoder pipeline, the proposed framework decouples spatial feature extraction from contextual refinement through a dedicated two-stage cascaded design. To the best of our knowledge, this is the truly innovative study conducted to systematically evaluate all 12 combinations arising from three UNet base variants (B1–B3) and four transformer-tuner modules (T1–T4) for carotid ultrasound segmentation, providing a comprehensive understanding of the performance–complexity trade-off in this domain.

The key contributions of this work are as follows: (1) a novel modular two-stage cascaded framework that decouples boundary-level feature extraction from contextual refinement, enabling independent optimization of each stage; (2) an exhaustive 12-model evaluation supported by multi-metric benchmarking across segmentation accuracy, AUC, and error reduction; (3) cross-institutional, cross-ethnic generalization validated across Japanese diabetic and Hong Kong post-menopausal cohorts without model retraining; and (4) K-parameter sensitivity and memorization-versus-generalization analyses that are rarely reported in the cIMT segmentation literature.

Based on experimental evidence, we make the following claims: tuner-enhanced models consistently outperform their standalone base counterparts across all evaluated metrics, the framework generalizes to unseen ethnic and demographic cohorts, and it achieves superior performance compared to nine benchmarked methods (Table 8). Quantitatively, the proposed tuner-augmented architectures yielded a 43.08% reduction in MA error, a 37.04% reduction in LI error, and a 23.99% reduction in IMT error relative to the base models alone. These gains, combined with a favourable accuracy-to-parameter ratio, confirm that the two-stage design offers meaningful clinical and computational advantages.

### 11.1. Benchmarking

As depicted in Table 8, the benchmarking comparison includes various studies (R1–10) and the suggested method (R10), covering single-stage Deep Learning (SDL), attention-based Deep Learning (ADL), and transformer-based Deep Learning (TDL). The assessment includes the number of rows in the dataset (DS), lumen–intima error (LIE), media–adventitia error (MAE), and intima media thickness error (cIMTE), total plaque area (cTPA), Jaccard index (cJI), Dice similarity (cDS), precision (cPrec), recall (cRec) and F1-score, allowing us to analyze the performance comprehensively based on each of the metrics.

### 11.2. Strengths, Weaknesses, and Extensions

The two-stage design allows for combining any UNet type with transformer-based tuners, facilitating the learning of fine detail spatial information and global contextual information. It has performed well in terms of segmentation accuracy, cross-validation, and generalization augmented across different sets and has an acceptable accuracy-to-parameter ratio. It is also flexible in various medical imaging tasks since it has a modular design [56].

The architecture of the model introduces multiple stages, which introduce greater computational demands and may limit the use of the model on lower end systems. Tuners that are derived from transformers can be very powerful, but they sacrifice interpretability [37]. Furthermore, the performance on different unseen data sets should be evaluated to assess its generalizability. Future research might lean towards lightweight transformer modules to keep overhead low, add explainability tools to build clinical trust, and use domain adaptation to achieve greater applicability. The real-time deployment of the model pruning methods is possible. A further limitation concerns reproducibility. The segmentation and measurement pipeline is implemented in software proprietary to AtheroPoint™ (Roseville, CA, USA), and the two training cohorts (DB1, Toho University, Japan; DB2, Chinese University of Hong Kong) are governed by institutional Ethics approvals and data-sharing agreements that do not permit public release. Consequently, the exact implementation and the primary training data cannot be distributed, and independent reproduction of the reported values is not possible from these sources alone. We have sought to mitigate this constraint in three ways: complete layer-level specifications of all three base networks and four tuner modules are given in Appendix B; all training settings, augmentation operations, and optimization parameters are reported in Section 3.5; and external validation was conducted on the openly licenced CUBS database, which is available to any investigator. Results obtained on the two proprietary cohorts should be interpreted as internally validated rather than independently replicated, and confirmation by external groups on open datasets remains necessary before clinical deployment.

## 12. Conclusions

In conclusion, the proposed two-stage transformer-tuner framework improved the accuracy, robustness, and cross-cohort generalization of carotid ultrasound segmentation by integrating convolutional encoder–decoder architectures with transformer and attention-based refinement. The framework was validated on multi-institutional cohorts and outperformed the corresponding single-stage methods across segmentation, efficiency, and measurement-level metrics. Its scalability, measurement precision, and modular adaptability indicate a viable pathway towards clinically deployable cardiovascular risk assessment, subject to prospective validation on larger and more diverse populations.

## Figures and Tables

**Table 1:** Base model evaluation.

Effect of Augmentation on Base UNets			
Type	w/o Augu (%)	w/ Augu (%)	Increase (%)
Base 1	78.65	96.41	22.58
Base 2	80.25	95.38	18.85
Base 3	84.63	92.44	9.23

Augu: Augmentation Increase: Performance Increase (measured on Dice Similarity).

**Table 2:** Effect of augmentation on the tuner.

Effect of Augmentation on B3 + T4			
Type	w/o Augu (%)	w/ Augu (%)	Increase (%)
ACC (%)	98.65	99.41	0.77
PREC (%)	79.96	91.33	14.2
REC (%)	84.63	90.04	6.39
F1 (%)	81.85	90.68	10.7

Acc: Accuracy, Prec: Precision, Rec: Recall, F1: F1 Score.

**Table 3:** Effect of K-Parameter.

Cross-Validation Results on Base 3 + Tuner 4							
Parameter	K2	K3	K4	K5	K10	TT	KM5
Base 3 + Tuner 4							
Acc (%)	99.39	99.47	99.23	99.4	99.50	99.99	99.22
Prec (%)	89.89	88.83	89.71	93.9	92.74	99.98	90.51
Rec (%)	89.73	93.60	85.54	87.55	90.59	99.97	84.15
F1 (%)	89.11	91.15	87.57	90.12	91.65	99.98	87.24
JI (%)	81.51	83.75	77.90	82.85	84.59	99.96	77.37
DS (%)	89.81	91.15	87.57	90.62	91.65	99.98	87.24
Loss	0.117	0.1043	0.2651	0.0907	0.0917	0.0445	0.2710

Acc: Accuracy, Prec: Precision, Rec: Recall, F1: F1 Score, JI: Jaccard Index, DS: Dice Similarity.

**Table 4:** Effect of tuner stage.

Performance of 4 Tuners on 3 Bases								
Config	Acc (%)	Prec (%)	DS (%)	JI (%)	TPA (mm^2^)	LIe (mm)	MAe (mm)	IMTe (mm)
**Effect of 4 Tuners on Base 1**								
B1 + T1	98.50	92.13	92.001	85.19	5.73	0.035	0.029	0.020
B1 + T2	99.25	89.55	90.281	82.48	5.95	0.030	0.034	0.030
B1 + T3	99.76	88.58	89.77	81.45	6.20	0.035	0.027	0.024
B1 + T4	99.84	87.50	87.52	81	6.23	0.034	0.035	0.019
**Effect of 4 Tuners on Base 2**								
B2 + T1	99.59	92.60	93.52	87.84	6.03	0.034	0.030	0.019
B2 + T2	99.72	93.22	94.52	89.84	3.83	0.033	0.031	0.019
B2 + T3	99.76	89.76	89.87	81.63	5.87	0.034	0.028	0.019
B2 + T4	99.12	90.12	85.59	80.79	3.12	0.031	0.027	0.021
**Effect of 4 Tuners on Base 3**								
B3 + T1	99.20	92.13	92.20	85.19	3.13	0.033	0.029	0.020
B3 + T2	99.23	93.45	90.80	83.76	6.00	0.036	0.029	0.018
B3 + T3	99.81	91.09	92.14	85.82	5.70	0.026	0.026	0.017
B3 + T4	99.89	96.01	94.14	88.71	5.71	0.021	0.022	0.014

Acc: Accuracy, Prec: Precision, JI: Jaccard Index, DS: Dice Similarity, TPA: Total Plaque Area, LIe: Lumen-intima error, MAe: Media-Adventia error, IMTe: Intima-Media Thickness Error.

**Table 5:** Seen vs. unseen analysis.

Models	DS (%)		JI (%)		cIMTe	
	Seen	Unseen	Seen	Unseen	Seen	Useen
F1: B1 + T1	92.00	88.74	85.19	80.92	0.020	0.027
F2: B1 + T2	90.28	87.05	82.48	78.13	0.030	0.038
F3: B1 + T3	89.77	86.42	81.45	77.06	0.024	0.031
F4: B1 + T4	87.52	84.18	81.00	76.55	0.019	0.026
F5: B2 + T1	93.52	90.31	87.84	83.40	0.019	0.026
F6: B2 + T2	94.52	91.47	89.84	85.62	0.019	0.025
F7: B2 + T3	89.87	86.55	81.63	77.28	0.019	0.026
F8: B2 + T4	85.59	82.41	80.79	76.13	0.021	0.029
F9: B3 + T1	92.20	89.03	85.19	80.94	0.020	0.027
F10: B3 + T2	90.80	87.66	83.76	79.22	0.018	0.025
F11: B3 + T3	92.14	88.97	85.82	81.30	0.017	0.024
F12: B3 + T4	94.14	91.05	88.71	84.39	0.014	0.021

JI: Jaccard Index, DS: Dice Similarity, cIMTe: Carotid Intima-Media Thickness Error.

**Table 6:** State-of-the-art comparisons.

BT	SN	PMA	M1	M2	M3	M4	M5	M6	M7	M8 *(Ours)*
**BT-1**	1	DS	0.91	0.85	0.82	0.93	0.89	0.93	0.91	**0.94**
	2	JI	0.84	0.76	0.70	0.87	0.81	0.87	0.88	**0.88**
	5	BA Plot	−0.65	−0.65	0.18	−0.57	−0.44	−0.42	−0.4	**−0.01**
**BT-2**	6	TrgTi (Mins)	1.54	**1.1**	16.3	7.9	8.15	8.3	8.5	14.3
	7	P# (Millons)	1.9	0.7	25.8	6.43	105	105.5	120	**247.1**
	8	S# (Mb)	7.40	**5.40**	98.8	24.52	4960	4964	5040	5892
	9	Energy usage (joules)	42	**18**	50	75	56	62	68	**214**
	10	GFLOPs	22.7	16.3	26.3	38.2	**15.9**	17.8	18.9	25.21
	11	Latency (ms)	1.6	**0.7**	2.4	6.25	3.312	3.326	3.56	7.51
**BT-3**	13	LIE (mm)	0.1268	0.077	0.098	0.0317	0.0394	0.0352	0.0369	**0.021**
	14	MAE (mm)	0.1342	0.077	0.1006	0.0331	0.0403	0.0343	0.0389	**0.022**
	15	cIMTE (mm)	0.132	0.124	0.0656	0.0224	0.0238	0.0225	0.0221	**0.014**
	16	PAE (mm^2^)	0.86	0.85	**0.52**	0.71	0.593	0.58	0.55	0.57

**BT:** Buffer type, **BT-1:** Buffer one (Performance), **BT-2:** Buffer two (Efficiency), **BT-3:** Buffer three (Measurement), **PMA:** Performance metrics attributes, **M1:** UNet, **M2:** DL Method, **M3:** Attention Unet, **M4:** NAG-Net, **M5:** TransUNet, **M6:** SwinUNet, **M7:** SSwAttSkip-XmerBot-UNet, **M8:** B3 + T4, **LIE:** Lumen–Intima Error, **MAE:** Media-Adventita Error, **cIMTE:** Carotid Intima-Media Thickness error, **PAE:** Plaque Area error, **BA Plot:** Bland-Altmann Plot, **JI:** Jaccard Index, **DS:** Dice similarity, **TrgTi:** Training time, **P#:** Number of trainable parameters, **S#:** Model size.

**Table 7:** Fusion models on statistical tests.

Pairwise Statistical Analysis of Different Model Configurations								
SN	Fusion A	Fusion B	95% CI of Δ	Cohen’s *d*	*t*-Test	MW U	Wilcoxon	HB
1	Base 1 + Tuner 1	Base 1 + Tuner 2	[−0.0052, −0.0008]	−0.092	*p* < 0.008	*p* < 0.065	*p* < 0.055	0.0045
2	Base 1 + Tuner 2	Base 1 + Tuner 3	[−0.0055, −0.0007]	−0.086	*p* < 0.009	*p* < 0.009	*p* < 0.009	0.0043
3	Base 1 + Tuner 3	Base 1 + Tuner 4	[−0.0058, −0.0008]	−0.088	*p* < 0.009	*p* < 0.007	*p* < 0.003	0.0040
4	Base 1 + Tuner 4	Base 2 + Tuner 1	[−0.0061, −0.0011]	−0.084	*p* < 0.007	*p* < 0.009	*p* < 0.008	0.0037
5	Base 2 + Tuner 1	Base 2 + Tuner 2	[−0.0050, −0.0008]	−0.085	*p* < 0.008	*p* < 0.008	*p* < 0.007	0.0035
6	Base 2 + Tuner 1	Base 2 + Tuner 3	[−0.0066, −0.0014]	−0.076	*p* < 0.006	*p* < 0.002	*p* < 0.007	0.0032
7	Base 2 + Tuner 1	Base 2 + Tuner 4	[−0.0079, −0.0017]	−0.075	*p* < 0.005	*p* < 0.006	*p* < 0.010	0.0030
8	Base 2 + Tuner 1	Base 3 + Tuner 1	[−0.0086, −0.0012]	−0.072	*p* < 0.009	*p* < 0.006	*p* < 0.055	0.0029
9	Base 3 + Tuner 1	Base 3 + Tuner 2	[−0.0049, −0.0007]	−0.069	*p* < 0.008	*p* < 0.007	*p* < 0.008	0.0025
10	Base 3 + Tuner 1	Base 3 + Tuner 3	[−0.0060, −0.0010]	−0.065	*p* < 0.007	*p* < 0.006	*p* < 0.008	0.0023
11	Base 3 + Tuner 1	Base 3 + Tuner 4	[−0.0072, −0.0012]	−0.062	*p* < 0.007	*p* < 0.006	*p* < 0.008	0.002

**Table 8:** Benchmarking table.

C0	C1	C2	C3	C4	C5	C6	C7	C8	C9	C10	C11	C12
SN	Citation	DLType	DS	cLIE (mm)	cMAE (mm)	cIMTE (mm)	cTPAe (mm)	cJI (%)	cDS (%)	cPrec (%)	cRec (%)	cF1 (%)
R1	Biswas et al. [50]	SDL	396	0.07 ± 0.01	0.077 ± 0.01	0.124 ± 0.01	-	-	-	-	-	-
R2	Biswas et al. [51]	SDL	250	-	-	0.093 ± 0.63	-	-	-	-	-	-
R3	Zhou et al. [52]	SDL	497	-	-	-	0.44 ± 4.05	-	88.	-	-	-
R4	Jain et al. [53]	SDL	379	-	-	-	-	79.63 ± 10.2	88.23 ± 7.3	89.05 ± 8.0	-	-
R5	Jain et al. [54]	ADL	1649	-	-	-	-	81.86 ± 5.90	86.5 ± 5.94	92.7 ± 7.20	-	-
R6	Huang et al. [29]	ADL	800	-	-	-	-	81.69	89. 92	-	-	-
R7	Yuan et al. [55]	TDL	1100	-	-	-	-	*-*	81.40 ± 0.06	91.1 ± 0.04	91.0 ± 0.03	91.3 ± 0.01
R8	Sarmun et al. [35]	TDL	1388	-	-	-	-	75.23 ± 0.85	85.86 ± 0.5	84.14 ± 1.4	-	-
R9	Singh et al. [38]	TDL	400	0.035	0.034	0.022	0.58	87.5	93. 31	94. 4	92.4	93.6
R10	Proposed (B3 + T4)	TDL	2876	0.021	0.022	0.014	0.57	88.7	94. 14	96. 09	95.04	94.1

(C0–C12): Indicates the number of columns. (R1–R12): Indicates the number of rows. SN: Serial number (R refers to row number). DL type: Deep learning type (SDL: Single stage dl; ADL: Attention-based dl; TDL: Transformer-based dl). cLIE: Carotid Lumen–Intima error. cMAE: Carotid media–adventitia error. DS: Dataset Size. cIMTE: Carotid Intima media thickness error. cTPAe: Carotid Total Plaque Area Error. cJI: Carotid Jaccard Index. cDS: Carotid Dice Similarity. cPrec: Carotid Precision. cRec: Carotid Recall. cF1: Carotid F1 Score.

## Data Availability

The external validation dataset (CUBS) is publicly available at https://data.mendeley.com/datasets/fpv535fss7/1 (accessed on 4 August 2026) under a CC BY 4.0 licence. The Japanese (DB1) and Hong Kong (DB2) cohorts used for training and internal validation are not publicly available, as they are subject to institutional Ethics approvals and data-sharing agreements that preclude redistribution; de-identified data may be made available from the corresponding author on reasonable request and subject to approval by the originating institutions. The segmentation software and associated scripts are proprietary to AtheroPoint™, Roseville, CA, USA, and are not publicly available; architectural specifications sufficient for reimplementation are provided in Appendix B.

## Supplementary Materials

The following supporting information can be downloaded at: https://www.mdpi.com/article/10.3390/diagnostics16162538/s1, Algorithm S1: Cascaded Base–Tuner Fused Segmentation (overview).

## Appendix A

**Table A1:** Acronym Table.

SN	Abbreviations	Definition
1	CVD	Cardiovascular diseases
2	DL	Deep learning
3	cIMT	Carotid Intima-Media Thickness
4	AE	AtheroEdge
5	AI	Artificial Intelligence
6	B1	UNet-based Base 1
7	B2	UNet-based Base 2
8	B3	UNet-based Base 3
9	T1	Transformer Tuner 1
10	T2	Transformer Tuner 2
11	T3	Transformer Tuner 3
12	T4	Transformer Tuner 4
13	IMTv	Intima-media thickness variability
14	CAD	Coronary-artery-disease
15	MRI	Magnetic-resonance-imaging
16	CT	Computed tomography
17	IVUS	Intravascular ultrasound
18	LIMA	Lumen-intima Media-Adventia
19	PA	Plaque Area
20	ROIs	Region of Interests
21	MLP	Multi-Layer Perceptron
22	MSA	Multihead Self-Attention
23	HDL	Hybrid Deep Learning
24	CNN	Convolution Neural Network
25	CUP	Cascaded Upsampler
26	SoloDL	Single stage deep
27	AttDL	Attention-based deep learning
28	XmerDL	Transformer-based deep learning

## Appendix B

### Appendix B.1. Architecture of Base 1

#### Appendix B.1.1. Overview and Motivation

Figure 1b: U-Net is a fully convolutional encoder–decoder network with a symmetric “U”-shaped topology. It was designed for biomedical image segmentation, where annotated data are scarce, and it introduced the now-standard use of skip connections that concatenate high-resolution encoder features with the decoder to recover the spatial detail lost during down-sampling. The network is trained end-to-end from very few images by relying heavily on data augmentation (notably elastic deformations) and an *overlap-tile* strategy that allows seamless segmentation of arbitrarily large images. The architecture has two symmetric halves: a contracting path (encoder) that captures context and progressively increases the semantic level while reducing spatial resolution, and an expansive path (decoder) that enables precise localization by up-sampling and fusing encoder features.

#### Appendix B.1.2. Contracting Path (Encoder)

The contracting path follows the typical architecture of a convolutional network. Each resolution level consists of the repeated application of: two 3×3 convolutions (unpadded/“valid” convolutions in the original paper), each followed by a rectified linear unit (ReLU); a 2×2 max-pooling operation with stride 2 for down-sampling. At each down-sampling step the number of feature channels is doubled. Starting from 64 channels, the encoder progresses through 64 → 128 → 256 → 512, reaching 1024 channels at the bottleneck. Using the original 572×572 input tile, the spatial resolution contracts accordingly (e.g., 568×568→284×284→⋯→28×28 at the bottleneck). Because valid convolutions discard border pixels, the feature-map size shrinks by 2 pixels after every 3×3 convolution.

#### Appendix B.1.3. Expansive Path (Decoder)

Every step in the expansive path consists of: An up-convolution (transposed/“up” convolution) with a 2×2 kernel that up-samples the feature map and halves the number of channels; A concatenation with the correspondingly cropped feature map from the contracting path (the skip connection); two 3×3 convolutions, each followed by a ReLU.

The cropping is necessary because, with unpadded convolutions, every encoder feature map is larger than its decoder counterpart; the central region is retained so the two can be concatenated. The decoder mirrors the encoder, halving channels at each level (1024 → 512 → 256 → 128 → 64) while doubling spatial resolution.

#### Appendix B.1.4. Skip Connections

The defining feature of U-Net is the direct, same-scale skip connection: the encoder feature map at level i is concatenated to the decoder feature map at the same level. These connections re-inject fine-grained, high-resolution information that is otherwise lost through pooling, allowing the decoder to produce sharp, well-localized boundaries. This “plain” skip connection is precisely the component that UNet++ and UNet 3+ later redesign.

#### Appendix B.1.5. Final Layer and Objective

A final 1×1 convolution maps each 64-component feature vector to the desired number of classes, followed by a pixel-wise soft-max. The network has 23 convolutional layers in total. The energy function is computed by a pixel-wise soft-max over the final feature map combined with a cross-entropy loss:

(A1) $$E=\sum_{x\in \Omega }w\left(x\right)\;log\left(p_{l\left(x\right)}\left(x\right)\right),$$

where $p_{l\left(x\right)}\left(x\right)$ is the soft-max probability of the true label $l\left(x\right)$ at pixel x, and $w\left(x\right)$ is a pre-computed weight map. The weight map compensates for class-frequency imbalance and, crucially, assigns large weights to the thin background regions separating touching objects, forcing the network to learn the separating borders:

(A2) $$w\left(x\right)=w_{c}\left(x\right)+w_{0}\cdot exp\;\left(-\frac{(d_{1}\left(x\right)+d_{2}\left(x\right){)}^{2}}{2\sigma^{2}}\right),$$

where $w_{c}$ is the class-balancing weight and $d_{1}$, $d_{2}$ are the distances to the nearest and second-nearest cell borders.

Practical note. The original network uses valid convolutions, hence the input (572×572) and output (388×388) differ in size. Modern implementations including the one used in this study replace valid with *same* (padded) convolutions so that input and output share spatial dimensions and cropping is no longer required. Batch normalization, absent from the 2015 formulation, is also commonly inserted after each convolution.

### Appendix B.2. Architecture of Base 2

#### Appendix B.2.1. Re-Designed (Nested, Dense) Skip Pathways

Figure 1c: Instead of connecting each encoder node directly to its decoder counterpart, UNet++ inserts a dense convolution block along every skip pathway. The number of convolution layers in a block depends on the pyramid level, so that the encoder feature map is progressively transformed until it is semantically closer to the decoder feature map it will be fused with. Within each pathway, dense connections (in the DenseNet sense) accumulate all previous feature maps: every node receives the outputs of all earlier nodes on the same skip pathway plus one up-sampled feature map from the denser block one level below.

#### Appendix B.2.2. Mathematical Formulation

Let $x^{i,j}$ denote the output of node $X^{i,j}$, where i indexes the down-sampling layer along the encoder and j indexes the convolution layer of the dense block along the skip pathway. The stack of feature maps at $x^{i,j}$ is computed as:

(A3) $$x^{i,j}=\left\{\begin{array}{ll} H\;\left(x^{i-1,j}\right), & j=0,\\ H\;\left(\left[\;[x^{i,k}{]}_{k=0}^{j-1},\;U\;\left(x^{i+1,j-1}\right)\right]\right), & j>0, \end{array}\right.$$

where $H\left(\cdot \right)$ is a convolution operation followed by an activation function, $U\left(\cdot \right)$ is an up-sampling layer, and $\left[\;\cdot \;\right]$ denotes channel-wise concatenation.

The formula has an intuitive reading: Nodes at level j=0 receive only a single input—the down-sampled output of the previous encoder layer. These nodes form the original U-Net encoder backbone. Nodes at level j>0 receive j+1 inputs: the outputs of the j previous nodes on the *same* skip pathway (same i), plus one up-sampled output from the lower-level, denser node $x^{i+1,j-1}$.

This nesting means that the top-row nodes $X^{0.1},X^{0.2},X^{0.3},X^{0.4}$ successively aggregate multi-depth features at full resolution.

#### Appendix B.2.3. Deep Supervision and Pruning

Because the nested design produces full-resolution feature maps at multiple nodes ($X^{0.1},\ldots ,X^{0.4}$), UNet++ attaches a segmentation head a 1×1 convolution followed by a sigmoid to each of them. This deep supervision enables two inference modes: Accurate mode, in which the segmentation maps from all branches are averaged; and Fast mode, in which the final map is taken from a single branch.

Selecting an early branch amounts to pruning the deeper part of the network at inference time, trading a small accuracy drop for a substantial reduction in parameters and inference latency. The pruned models are denoted $\mathrm{UNet}++\;L^{d}$, where d is the retained depth.

#### Appendix B.2.4. Objective Function

A hybrid loss combining binary cross-entropy and the Dice coefficient is applied to each of the semantic levels:

(A4) $$L\left(Y,\hat{Y}\right)=-\frac{1}{N}\sum_{b=1}^{N}\left(1/2\;Y_{b}\;log{\hat{Y}}_{b}\;+\;\frac{2\;Y_{b}\;{\hat{Y}}_{b}}{Y_{b}+{\hat{Y}}_{b}}\right),$$

where $Y_{b}$ and ${\hat{Y}}_{b}$ are the ground-truth label and predicted probability of the b-th pixel, and N is the number of pixels in a batch. Under deep supervision the loss is summed (or averaged) over all four segmentation branches.

### Appendix B.3. Architecture of Base 3

Figure 1d: UNet++ enriches the skip pathways but still fuses feature maps drawn from restricted scales it does not explicitly exploit the *full* range of scales available in the network. UNet 3+ addresses this with two contributions: full-scale skip connections, which combine low-level detail with high-level semantics from feature maps at *every* scale, and full-scale deep supervision, which learns hierarchical representations from the aggregated feature maps. A classification-guided module and a hybrid loss further sharpen organ boundaries and suppress false positives, while the overall parameter count is *reduced* relative to UNet++.

#### Appendix B.3.1. Full-Scale Skip Connections

Consider a five-stage network with encoder stages $E^{1},\ldots ,E^{5}$ and decoder stages $D^{1},\ldots ,D^{5}$ ($E^{5}$ serving as the bottleneck). Unlike U-Net, where a decoder stage receives only the same-scale encoder map, each UNet 3+ decoder stage $D^{i}$ aggregates feature maps from all scales.

Smaller-scale (shallower) encoder stages $E^{1},\ldots ,E^{i-1}$ carry fine-grained, low-level detail. They are down-sampled to the resolution of stage i using non-overlapping max pooling.

The same-scale encoder stage $E^{i}$ is passed through directly. Larger-scale (deeper) decoder stages $D^{i+1},\ldots ,D^{5}$ carry coarse-grained, high-level semantics. They are up-sampled to the resolution of stage i using bilinear interpolation.

Each of these feature maps is passed through 64 convolution filters of size 3×3 to *unify the channel dimension to 64*. The five unified feature maps (in the five-stage example) are then concatenated, giving 5×64=320 channels, and fused by a feature-aggregation step of 320 filters of size 3×3, followed by batch normalization and a ReLU.

#### Appendix B.3.2. Mathematical Formulation

Let $X_{De}^{i}$ denote the i-th decoder feature map. Using the operators $C\left(\cdot \right)$ for a 3×3 convolution, $D\left(\cdot \right)$ for down-sampling by max pooling, $U\left(\cdot \right)$ for bilinear up-sampling, $\left[\cdot \right]$ for concatenation, and $A\left(\cdot \right)$ for the aggregation “conv–BN–ReLU” operation, the third decoder stage $X_{De}^{3}$ in a five-stage network is:

(A5) $$X_{De}^{3}=A\;\left(\left[\;\underset{\mathrm{smaller}\text{-}\mathrm{scale}\;\mathrm{encoders}}{\underset{⏟}{C\left(D\left(X_{En}^{1},4\right)\right),\;C\left(D\left(X_{En}^{2},2\right)\right)}},\;\underset{\mathrm{same}\;\mathrm{scale}}{\underset{⏟}{C\left(X_{En}^{3}\right)}},\;\underset{\mathrm{larger}\text{-}\mathrm{scale}\;\mathrm{decoders}}{\underset{⏟}{C\left(U\left(X_{De}^{4},2\right)\right),\;C\left(U\left(X_{De}^{5},4\right)\right)}}\;\right]\right)$$

where the integer argument of D and U is the (down/up)-sampling factor. Every decoder stage is therefore composed of N=5 scale contributions of 64 channels each, i.e., 320 channels per decoder stage. Because the channel width is fixed at 64 per scale (rather than growing with dense blocks as in UNet++), UNet 3+ achieves full-scale fusion with *fewer* parameters than UNet++.

#### Appendix B.3.3. Full-Scale Deep Supervision

To learn hierarchical representations from the full-scale aggregated feature maps, UNet 3+ applies deep supervision at every decoder stage. The last layer of each decoder stage is passed through a 3×3 convolution, bilinearly up-sampled to the full input resolution, and finally through a sigmoid to yield a side-output segmentation map that is compared against the ground truth. This differs from the deep supervision of UNet++, which is generated from full-resolution feature maps at the top nodes only.

In many volumetric datasets, most slices contain no target organ, and shallow decoder stages tend to over-segment such slices, producing false positives. To correct this, UNet 3+ adds a classification-guided module that predicts whether an input contains the organ. The deepest feature map $X_{En}^{5}$ is passed through:

$$X_{En}^{5}\;\to \;\mathrm{Dropout}\;\to \;{\mathrm{Conv}}_{1\times 1}\;\to \;\mathrm{AdaptiveMaxPool}\;\to \;\mathrm{Sigmoid}\;\to \;argmax,$$

yielding a binary indicator (organ present/absent). This {0,1} scalar is multiplied with each side-output segmentation map, so that slices classified as organ-free are zeroed out. The classification branch is optimized with a binary cross-entropy loss and acts as a spatial regularizer that guides the segmentation output.

### Appendix B.4. Architecture of Tuner 1

The following part of this subsection explains the design of Figure 2a: M6:SSwAttSkip-XmerBot-UNet. A grayscale digital image (y) with dimensions H × W × C presents spatial resolution H × W (height, width) and a total of C channels. The primary goal is to create binary pixel maps that have the same H × W dimensions. Images get abstractly encoded through standard CNNs like UNet before generating entire resolution outputs through model decoding functions. The application of transformers in traditional encoder architecture enables our system to execute self-attention systems. Attention-based Skip connections enable the model to selectively focus on relevant spatial features while suppressing irrelevant activations. Encoder wing as transformer describes direct transformer implementation for image patch encoding, but Transformer in UNet and attention in skip connections examines the single stage with attention in skip connection and transformer in the bottle neck layer (M6:SSwAttSkip-XmerBot-UNet) architecture.

#### Mathematical Representation and Data Flow of Transformer in the Bottleneck

The input x gets tokenized through reshaping to form a sequence of flattened 2D patches with patches of size P × P and $N=\frac{HW}{P^{2}}$ defining the number of image patches as the input sequence length.Patch Embedding. The vectorized patches $y_{p}$ get transformed into a latent D-dimensional embedding space through a trainable linear projection. Learning specific position embeddings gets applied to the patch embeddings because it enables retention of positional information through this process:

(A6) $$Z0{=[y\;}_{p}^{1}E,\;{y\;}_{p}^{2}E\ldots \ldots .{y\;}_{p}^{n}E]+Ep\;$$

here E ∈ $R^{{(P}^{2}.C)\times D}$ is the patch embeded projection, and Ep ∈ $R^{N\times D}$ represents the embedded position. The encoder of transformer have L layers of Multihead Self-Attention (SA) and Multi-Layer Perceptron (LP) blocks. Therefore the output of the *l*th block can be written as:

(A7) $$O_{l}^{\prime }=SA\left(LN\left(O_{l}-1\right)\right)+O_{l-1},$$

(A8) $$O_{l}=LP\left(LN\left(O_{l}^{\prime }\right)\right)+O_{l}^{\prime }$$

where LN(·) represents the normalization layer and $O_{l}$ is the image representation (encoded). The Transformer projection layer is illustrated in Figure 2a. An intuitive method for segmentation delivers the up sampled ${Z_{l}\in R}^{\frac{HW}{P^{2}}\times D}$ to its full resolution so a dense output can be predicted. To restore the spatial organization of $Z_{l}$, must first undergo a size change from $\frac{HW}{P^{2}}$ to $\frac{H}{P}\;\times \frac{W}{P}$. We use a 1 × 1 convolution. The resized feature channels are trimmed down to match the class numbers, while a direct bilinear upsample operation returns predictions to H × W image resolutions. We refer to the basic version of upsampling without innovation as “None” in our decoder design. The combination of Transformers with basic upsampling produces satisfactory results, but sub-optimal compared to their potential in segmentation work, since anticipating $\frac{H}{P}\;\times \frac{W}{P}$ produces smaller output than H × W, which leads to information loss of essential organ shapes and boundaries. The M6:SSwAttSkip-XmerBot-UNet architecture combines CNN and Transformer networks in its encoder section while implementing an up-sampling cascade to achieve accurate positioning. The proposed M6:SSwAttSkip-XmerBot-UNet architecture can be observed in Figure 3. M6:SSwAttSkip-XmerBot-UNet uses a hybrid model of CNN along with Transformer architecture as in its bottleneck but it begins by utilizing CNN for feature extraction of input data. The CNN feature map undergoes patch embedding for 1 × 1 patches instead of raw images. This architectural design enables us to use the CNN intermediate feature maps during decoding and provides better performance than a pure Transformer as an encoder. Our cascaded up sampler (CUP) combines successive up sampling passes to deduce the hidden feature which is subsequently used for creating the final segmentation mask. The hidden feature ${Z_{l}\in R}^{\frac{HW}{P^{2}}\times D}$ gets reshaped as $\frac{H}{P}\;\times \frac{W}{P}\times D$ before passing through CUP which consists of multiple upsampling blocks. The image upscaling process from $\frac{H}{P}\;\times \frac{W}{P}$ to H × W uses block structures that implement the sequence of a Spatial resolution is restored by 2× upsampling, then refined using a 3 × 3 convolution and ReLU nonlinearity. A U-shaped architecture has been constructed by combining CUP with the hybrid bottleneck transformer with encoder for performing feature aggregation through attention enhanced skip-connections at various resolution levels. The full CUP architecture design, along with its sequential attention skip-connections, appears in Figure 3.

### Appendix B.5. Architecture of Tuner 2

In this subsection, we discuss Figure 2b: the two-stage attention-guided network, the Nested attention-guided network (NAG-Net), proposed by Huang et al. [29], for simplicity, we term it as DSwAttDec-UNet (Double-stage with attention in Decoder-UNet) to explain the two-stage attention-enabled network for accurate segmentation of the LIMA in carotid ultrasound scans. Unlike the attention models. DSwAttDec-UNet includes the clinical knowledge attained through the dual-stage architecture which gradually enhances segmentation accuracy while reducing post-processing complexity.

The architecture of the two interconnected networks is termed as DSwAttDec-UNet Stage I, It segments the Intima media Region, and then the DSwAttDec-UNet Stage II segments the LIMA boundaries. Now DSwAttDec-UNet stage I subnetwork follows the UNet encoder–decoder architecture, where the encoder consisting of four convolution blocks followed by batch normalization, ReLU activation and max pooling layers (Kernel = 2), taking into account feature extraction of each encoder in sequence respectively, On the other hand, Binary Image Map (BIP) is created using segmentation features are reconstructed by the decoder and clinically important areas are marked on the image helping DSwAttDec-UNet Stage II to do the segmentation more accurately. This process is represented in Figure 2b, showing attention in the decoder path improves the model’s focus on arterial structures.

The DSwAttDec-Unet stage II sub-network employs a dual encoder structure, where one encoder process the carotid ultrasound scan grayscale image, and the second one processes the BIP form the DSwAttDec-UNet stage I, both of the encoders follow a VGG-16-enabled architecture, which extracts the multi-scale features that are fused using an Attention-based feature fusion block (FFB) This fusion block adaptively selects the most relevant features, for better boundary delineation while minimizing the noise Figure 2b, represent the two encoder structure, emphasizing the role of attention driven feature selection.

### Appendix B.6. Architecture of Tuner 3

Figure 2c demonstrates the overall architecture of the suggested SSSwin-UNet. M7: SSSwin-UNet: It is a network The model includes an encoder, a bottleneck module, a decoder, and inter-stage skip connections. Transformer block is a fundamental block of SSSwin-UNet. In the case of the encoder, the medical images are broken down into non-overlapping 4 × 4 patch size in order to encode the inputs into sequence embeddings.

Through this method of partition, the feature dimension of a given patch is reduced to 4 × 4 × 3 = 48. In addition, projected feature dimension is embedded in arbitrary with linear embedding. The patched tokens windows are transmitted over a few. To construct hierarchical representations using patch-merging layers and architecture is composed of Transformer blocks that focus on feature representation learning, while hierarchical patch-merging layers are employed to perform spatial down-sampling and increase the feature dimensionality. Our inspiration is U-Net and we create a decoder some of which is founded on a symmetrical transformer. In the proposed architecture, the Transformer module is not employed as a conventional decoder. Instead, its contextual representations are progressively multi-scale encoder features are incorporated through skip connections. This design choice mitigates the spatial information degradation introduced by successive down-sampling operations.

Feature resolution recovery is achieved through a dedicated patch-expansion mechanism, which differs fundamentally from the patch-merging operation used during encoding. The patch-expansion layer redistributes feature representations across neighbouring spatial dimensions, enabling hierarchical resolution restoration at multiple up-sampling stages. Specifically, intermediate layers perform resolution doubling, while the final expansion stage restores the feature map to the original spatial scale through a four-fold up-sampling operation. The reconstructed high-resolution representations are subsequently reshaped into dense feature maps, followed by a linear projection layer that generates pixel-wise segmentation outputs. Detailed descriptions of each architectural component are provided in the subsequent sections.

#### Appendix B.6.1. Transformer Architecture

This transformer is a multiheaded self-attention (SA) model, as compared to a traditional one with a single head. transformer block is made of shifted patched windows. Now, Figure 2, shows succeeding single-stage swined transformer block (SST). Every SST is made up of multiheaded self- attention module, Normalization (LN) layer, residual-layer and two layer perceptron (LP) with GELU with none linearity.

Multiheaded self-attention based window (W-SA) module and the multiheaded self-attention with shifted window (SW-SA) module is applied to the two consecutive SST respectively. By much window partitioning, (of type SSTs) can then be formulated as:

(A9) X^l = W−SA(LN(Xl−1))+Xl−1

(A10) Xl=LP(LN(X^l))+X^l,

(A11) X^l+1=SW−SA(LN(Xl))+Xl

(A12) Xl+1=LP(LN(X^l+1))+X^l+1 

here X^l+1 and $X^{l}$ output of (S)W-SA block and LP are represented *l*th block of the module, respectively. Just like the previous works, the self-attention is calculated as given below:

(A13) $$Att(Q^{\prime },K^{\prime },V^{\prime })=SM(\frac{QK^{T}}{\surd d}+B^{\prime })V^{\prime },$$

here Att represents Attention *SM* shows soft-max activation, Q^′^, K^′^, V^′^ represent the query, key, and value matrices in $\in R^{M^{2}\times d}.\;M^{2}$ and d where the number of patches is in a window and dimension of query or key, respectively. And the $B^{\prime }$ values via matrix of bias $B^{\prime }\in R^{\left(2M-1)\times (2M+1\right)}$.

#### Appendix B.6.2. Swin-Encoder (Single-Stage)

In the ssswin-encoder, tokenized inputs with C feature channels and spatial resolution $\frac{H}{4}\times \frac{W}{4}$ undergo representation learning through two successive Swin Transformer blocks, during which the feature size and spatial scale are preserved. This is followed by a patch-merging stage that halves the number of tokens and outputs feature maps with twice the original channel dimensionality.

This 1 repetition of process will be carried encoder-side patch aggregation is performed by grouping adjacent patches into four localized regions. The feature maps undergo spatial downsampling by a factor of two, followed by a concatenation operation that expands the channel dimension by four times. A linear transformation is then employed to project the concatenated features into a representation with twice the original feature dimensionality.

#### Appendix B.6.3. Bottleneck and Decoder

Transformer is so profound it cannot be converged and therefore can only be two consecutive Swin. Transformer blocks are used to learn. feature representation to construct the bottleneck. At the bottleneck stage, the representation maintains the same spatial scale and feature dimensionality as the encoder output. The decoder is designed symmetrically using Swin Transformer blocks and incorporates patch-expanding layers instead of patch-merging operations.

These layers rearrange local feature neighbourhoods to perform 2 × 2 spatial upsampling and project the features to half of their previous dimensionality. In the first patch-expanding stage, a linear layer projects the input tokens to a higher-dimensional space (W/32 × H/32 × 16C). A subsequent rearrangement operation up samples the spatial resolution by 2 × 2 and compresses the feature dimension to one-fourth of the expanded size, yielding W/16 × H/16 × 4C.

### Appendix B.7. Architecture of Tuner 4

#### Appendix B.7.1. Overall Architecture

Figure 2d: The central aim of our design is to embed a pyramidal hierarchy within the Transformer paradigm, enabling it to produce feature maps at several resolutions a property that dense prediction problems such as detection and semantic segmentation rely on. Figure 3 summarizes the network. As in conventional CNN backbones, the model is organized into four sequential stages, each yielding feature maps at a distinct scale. Every stage follows the same template: a patch-embedding module followed by a stack of *L*_i_ Transformer encoder blocks.

Consider the first stage. An input image of dimensions *H* × *W* × 3 is partitioned into *HW*/4^2^ non-overlapping patches, each spanning 4 × 4 × 3. These patches are flattened and pushed through a linear projection, producing embeddings of shape (*HW*/4^2^) × *C*_1_. A positional embedding is then added, and the combined sequence is processed by an encoder of *L*_1_ layers. The resulting sequence is folded back into a spatial feature map *F*_1_ of size (*H*/4) × (*W*/4) × *C*_1_. Repeating this procedure feeding each stage the output of the one before it—yields the remaining maps *F*_2_, *F*_3_, and *F*_4_, whose effective strides relative to the original image are 8, 16, and 32 pixels, respectively. The resulting pyramid {*F*_1_, *F*_2_, *F*_3_, *F*_4_} plugs directly into a wide range of downstream pipelines, including classification, detection, and segmentation.

#### Appendix B.7.2. Feature Pyramid for Transformer

Whereas CNN backbones obtain their multi-scale representations by varying convolutional stride, PVT relies instead on a *progressive shrinking* mechanism, in which the patch-embedding layers themselves govern how the feature-map resolution contracts from stage to stage. Let *P*_i_ be the patch size used at stage *i*. Entering that stage, the incoming feature map *F*_i−1_ of shape *H*_i−1_ × *W*_i−1_ × *C*_i−1_ is split evenly into (*H*_i−1_*W*_i−1_)/*P*_i_^2^ patches, and each patch is flattened and mapped to a *C*_i_-dimensional vector. Following this projection, the embedded sequence can be regarded as having spatial dimensions (*H*_i−1_/*P*_i_) × (*W*_i−1_/*P*_i_) × *C*_i_—that is, both height and width shrink by a factor of *P*_i_. Choosing *P*_i_ per stage therefore gives us direct, flexible control over the scale at every level, which is what makes a Transformer-based feature pyramid feasible.

#### Appendix B.7.3. Transformer Encoder

At stage *i*, the encoder comprises *L*_i_ identical blocks, each pairing an attention sublayer with a feed-forward sublayer. Because PVT must operate on high-resolution maps (as fine as 4-stride), we substitute a *spatial-reduction attention* (SRA) sublayer for the standard multi-head attention (MHA) used in the vanilla Transformer. Like MHA, SRA takes a query *Q*, a key *K*, and a value *V* and returns a refined representation. The distinction is that SRA compresses the spatial extent of *K* and *V* prior to the attention computation (Figure 4), which sharply lowers the compute and memory demands. Formally, SRA at stage *i* is expressed as:

(A14) $$SRA\left(Q,K,V\right)=Concat\left(head_{0},\ldots ,head_{N_{i}}\right)W^{O}$$

(A15) $$head_{j}=Attention\left(Q\;W_{j}^{Q},SR\left(K\right)W_{j}^{K},SR\left(V\right)W_{j}^{V}\right)$$

Here Concat(·) denotes concatenation, and *W*_j_^Q^, *W*_j_^K^, *W*_j_^V^ of shape *C*_i_ × *d*_head_, together with *W*^O^ of shape *C*_i_ × *C*_i_, are learnable projection matrices. With *N*_i_ heads at stage *i*, each head has dimension *d*_head_ = *C*_i_/*N*_i_. The operator SR(·), which collapses the spatial dimension of its input (either *K* or *V*), is defined as:

(A16) $$SR\left(x\right)=Norm\left(Reshape\left(x,R_{i}\right)W^{S}\right)$$

In this expression *x* of shape (*H*_i_*W*_i_) × *C*_i_ is the input sequence and *R*_i_ is the reduction ratio applied to attention at stage *i*. The operation Reshape(*x*, *R*_i_) recasts *x* into a sequence of shape (*H*_i_*W*_i_)/*R*_i_^2^ × (*R*_i_^2^*C*_i_), while *W*^S^ of shape (*R*_i_^2^*C*_i_) × *C*_i_ is a linear map that brings the channel dimension back down to *C*_i_. The term Norm(·) refers to layer normalization. Attention itself is computed exactly as in the original Transformer:

(A17) $$Attention\left(q,k,v\right)=Softmax\left(\frac{qk^{T}}{\sqrt{d_{head}}}\right)v$$

Taken together, these equations show that SRA cuts the attention operation’s compute and memory footprint by a factor of *R*_i_^2^ relative to MHA, allowing the module to accommodate substantially larger feature maps and sequences under a fixed resource budget.

## Appendix C

- Lumen-Intima Error (LIE):

This measures the absolute distance error between ground-truth and AI-predicted lumen–intima boundary.

(A18) $$Lumen-Intima\;Error\left(LIE\right)=\frac{1}{N}\sum_{i=1}^{N}\left|{LI}_{i}^{GT}-{LI}_{i}^{AI}\right|$$

where ${LI}_{i}^{GT}$ = Gold Standard lumen–intima distance (in mm), ${LI}_{i}^{AI}$ = AI-predicted lumen–intima distance (in mm).

- Media–Adventitia Error (MAE):

The Media–Adventitia Error (MAE) measures the absolute error between the ground-truth and AI-predicted media–adventitia boundary positions.

(A19) $$Media-Aventitia\;Error\left(MAE\right)=\frac{1}{N}\sum_{i=1}^{N}\left|{MA}_{i}^{GT}-{MA}_{i}^{AI}\right|$$

where ${MA}_{i}^{GT}$ = Gold Standard Media–Adventitia distance (in mm), ${MA}_{i}^{AI}$ = AI-predicted Media–Adventitia distance (in mm).

- Carotid Intima-Media Thickness (cIMT):

cIMT is computed as the absolute difference between the paired intima–media thickness (IMT) values obtained from the ground truth and the AI-predicted segmentations. Specifically, IMT is measured using a polyline distance–based method, where corresponding points along the intima and media–adventitia boundary polylines are paired, and the perpendicular distances between these polylines are calculated. The mean thickness is derived from these point-wise distances, and the cIMT is defined as the absolute difference between the ground truth and predicted mean IMT values.

(A20) $$Carotid\;Intima-Media\;Thickness\;Error\;{\left(cIMT\right)}_{Absolute}=\frac{1}{N}\sum_{i=1}^{N}\left|{\left(cIMT\right)}_{GT,i}-{\left(cIMT\right)}_{AI,i}\right|$$

where ${\left(cIMT\right)}_{GT,i}$ = cIMT distance (in mm) from gold standard, ${\left(cIMT\right)}_{AI,i}$ = AI-predicted cIMT distance (in mm).

- Plaque Area Error (PAE):

Plaque Area Error (PAE) evaluates the difference in plaque area estimation between the ground-truth and AI predictions.

(A21) $$Plaque\;Area\;Error\;Absolute\;{\left(PAE\right)}_{Absolute}=\frac{1}{N}\sum_{i=1}^{N}\left|{PA}_{i}^{GT}-{PA}_{i}^{AI}\right|$$

where ${PA}_{i}^{GT}$ = Ground-Truth Plaque Area (in mm^2^), ${PA}_{i}^{AI}$ = AI-predicted Plaque Area (in mm^2^).

- Dice Similarity Coefficient (DS):

It shows the degree of overlap between |cPred| and |cGT| segmentations, with higher scores indicating better model accuracy.

(A22) $$Dice\;Similarity\;(DS)=\frac{\left(2\times |cPred\cap cGT|\right)}{\left(|cPred|+|cGT|\right)}$$

where |cPred| represents the total number of pixels in the predicted segmentation, |cGT| represents the total number of pixels in the ground-truth segmentation |cPred∩cGT| denotes the number of pixels that are correctly classified as belonging to the segmented region in both the predicted and ground-truth masks (intersection).

- Jaccard Index (JI):

Jaccard index measures the similarity between predicted and ground-truth segmentations, with higher values indicating greater overlap and it is computed as:

(A23) $$Jaccard\;Index\;(JI)=\frac{\left|cPred\cap cGT\right|}{\left(|cPred|+|cGT|-|cPred\cap cGT|\right)}$$

where |cPred| represents the total number of pixels in the predicted segmentation, |cGT| represents the total number of pixels in the ground-truth segmentation, cPred∩cGT| denotes the number of pixels that are correctly classified as belonging to the segmented region in both the predicted and ground-truth masks (intersection).

- Bland Altmann (BA):

The GT and *p* values are then assessed as to the degree of agreement with one another through the plotting of their average values against their differences in the Bland–Altman plot and the assessment of systematic bias or variability, and can be given as:

(A24) $$Bland\;Altmann\;\left(BA\right)=M=\frac{\left|cPred+cGT\right|}{2}D=\left|cGT-cPred\right|$$

where M represents the Mean, D represents the Difference, |cPred| represents the total number of pixels in the predicted segmentation, |cGT| represents the total number of pixels in the ground-truth segmentation.
