# Supplementary material for: Twelve Fused Models by Fusing Four Types of Transformer-Based Tuners on Three Base UNet Architectures for Carotid Wall Segmentation and Plaque Burden/Intima-Media Thickness Measurements in Ultrasound Scans: A Scientific Validation Study
Source: Diagnostics (Basel). 2026 Aug 12;16(16):2538. doi: 10.3390/diagnostics16162538 (PMC13511551; doi:10.3390/diagnostics16162538)
Supplement: Supplementary file 1 [file diagnostics-16-02538-s001.zip › diagnostics-4426199-supplementary.pdf]

Algorithm S1: Cascaded Base–Tuner Fused Segmentation (overview)

Input: Base pool $\mathcal{B} = \{B_{\boldsymbol{\phi}}^{(m)}\}_{m=1}^{3}$; tuner pool $\mathcal{T} = \{T_{\boldsymbol{\psi}}^{(n)}\}_{n=1}^{4}$ (Table 1); **user-selected** indices $b \in \{1,2,3\}$, $t \in \{1,2,3,4\}$; subject-level splits $\mathcal{D}_{\text{tr}}, \mathcal{D}_{\text{val}}, \mathcal{D}_{\text{te}}$ (no leakage); threshold τ

Output: Fused model $F^{(b,t)} = T_{\boldsymbol{\psi}^\star}^{(t)} \circ \Pi \circ B_{\boldsymbol{\phi}^\star}^{(b)}$; test-metric summary $\mathcal{M}$

Select base $B_{\boldsymbol{\phi}}^{(b)}$ and tuner $T_{\boldsymbol{\psi}}^{(t)}$

`/* The pair` (b,t) `fixes one of the twelve configurations; the base supplies a coarse prior and the tuner refines its boundary. */`

Stage 1 (base). Train $B^{(b)}$ on $\mathcal{D}_{\text{tr}}$ under the objective in Eq. (1); retain checkpoint $\boldsymbol{\phi}^\star$ of best validation Dice

`/* The crude network learns a rough foreground map. Subject-level splits prevent leakage and only the best-validation state` $\boldsymbol{\phi}^\star$ `is kept. */`

Prior caching. Freeze $\boldsymbol{\phi}^\star$; form refiner inputs $x^+ = \Pi\big(x, \sigma(B^{(b)}(x;\boldsymbol{\phi}^\star))\big)$ once, reused by all tuners of base b

`/* Each prior is computed a single time and concatenated with its image. Caching lets the four tuners of base` b `share one prior set. */`

Stage 2 (tuner). Train $T^{(t)}$ on cached pairs $\{(x^+, y)\}$; retain $\boldsymbol{\psi}^\star$ of best validation Dice

`/* The transformer tuner sharpens the boundary from the coupled input` $[x \| \hat{p}]$`. The base is fixed, so only` $\boldsymbol{\psi}$ `is updated. */`

Test. Cascade $B^{(b)} \to T^{(t)}$ on $\mathcal{D}_{\text{te}}$, threshold at τ, post-process, and score vs. y

`/* Frozen base and tuner run in sequence; the refined map is binarised at` τ`, cleaned (largest connected component), and scored (Dice/IoU/F1/Prec). */`

return $F^{(b,t)}$, $\mathcal{M}$

Objective. Both stages minimise a composite region–boundary loss

$$\mathcal{L}(\hat{p}, y) = \lambda_{\text{bce}}\, \text{BCE}(\hat{p}, y) + \lambda_{\text{dice}}\big(1 - \text{Dice}(\hat{p}, y)\big), \tag{S1}$$

with $\sigma(\cdot)$ the logistic function and $\hat{p} = \sigma(\cdot)$ a per-pixel foreground probability.

Notation. $\mathcal{D} = \{(x_i, y_i)\}$ are image–mask pairs, $x \in \mathbb{R}^{H\times W}$ a grayscale ultrasound image and $y \in \{0,1\}^{H\times W}$ its annotation. A base $B_{\boldsymbol{\phi}}^{(b)}$ yields a crude prior $\hat{p} = \sigma(B_{\boldsymbol{\phi}}^{(b)}(x))$; a tuner $T_{\boldsymbol{\psi}}^{(t)}$ refines it; Π denotes the image–prior coupling operator.

Remark (twelve fused configurations). Sweeping the selector (b,t) over $\{1,2,3\} \times \{1,2,3,4\}$ instantiates all $|\mathcal{B}| \times |\mathcal{T}| = 12$ fused models. As Stage 1 depends only on b, each base is trained *once* and its cached priors are reused across the four tuners, so the full grid costs 3 base runs + 12 tuner runs rather than 12 end-to-end trainings.
